# The Phenylacetic Acid Catabolic Pathway Regulates Antibiotic and Oxidative Stress Responses in Acinetobacter

**DOI:** 10.1128/mbio.01863-21

**Published:** 2022-04-25

**Authors:** Anna J. Hooppaw, Jenna C. McGuffey, Gisela Di Venanzio, Juan C. Ortiz-Marquez, Brent S. Weber, Tasia Joy Lightly, Tim van Opijnen, Nichollas E. Scott, Silvia T. Cardona, Mario F. Feldman

**Affiliations:** a Department of Molecular Microbiology, Washington University School of Medicine in St. Louis, St. Louis, Missouri, USA; b Biology Department, Boston College, Chestnut Hill, Massachusetts, USA; c Department of Microbiology and Medical Microbiology, University of Manitoba, Winnipeg, Manitoba, Canada; d Department of Microbiology and Immunology, The Peter Doherty Institute for Infection and Immunity, University of Melbourne, Parkville, Victoria, Australia; University of Georgia

**Keywords:** *Acinetobacter*, stress response, antibiotics, gene regulation, phenylacetic acid

## Abstract

The opportunistic pathogen Acinetobacter baumannii is responsible for a wide range of infections that are becoming increasingly difficult to treat due to extremely high rates of multidrug resistance. Acinetobacter’s pathogenic potential is thought to rely on a “persist and resist” strategy that facilitates its remarkable ability to survive under a variety of harsh conditions. The *paa* operon is involved in the catabolism of phenylacetic acid (PAA), an intermediate in phenylalanine degradation, and is the most differentially regulated pathway under many environmental conditions. We found that, under subinhibitory concentrations of antibiotics, A. baumannii upregulates expression of the *paa* operon while simultaneously repressing chaperone-usher Csu pilus expression and biofilm formation. These phenotypes are reverted either by exogenous addition of PAA and its nonmetabolizable derivative 4-fluoro-PAA or by a mutation that blocks PAA degradation. Interference with PAA degradation increases susceptibility to antibiotics and hydrogen peroxide treatment. Transcriptomic and proteomic analyses identified a subset of genes and proteins whose expression is affected by addition of PAA or disruption of the *paa* pathway. Finally, we demonstrated that blocking PAA catabolism results in attenuated virulence in a murine catheter-associated urinary tract infection (CAUTI) model. We conclude that the *paa* operon is part of a regulatory network that responds to antibiotic and oxidative stress and is important for virulence. PAA has known regulatory functions in plants, and our experiments suggest that PAA is a cross-kingdom signaling molecule. Interference with this pathway may lead, in the future, to novel therapeutic strategies against A. baumannii infections.

## INTRODUCTION

Acinetobacter baumannii is an opportunistic pathogen that causes a wide range of infections, including bacteremia, ventilator-associated pneumonia, and urinary tract and wound infections (1, 2). A. baumannii is becoming increasingly difficult to treat, and currently about 60% of clinical isolates are multidrug resistant (MDR). Additionally, A. baumannii causes particularly recalcitrant infections due to its ability to withstand antibiotic treatment without designated antibiotic resistance genes (3–5). This is a great concern as surviving antibiotic treatment is known to facilitate the development of true antibiotic resistance (6). However, the mechanisms used by A. baumannii to adapt to and tolerate hostile conditions, such as antibiotic treatment, remain largely unknown.

Our lab has previously reported that A. baumannii ATCC 17978 (Ab17978) responds to subinhibitory concentrations of the antibiotic combination trimethoprim and sulfamethoxazole (TMP/SMX) by repressing expression of Csu pili, leading to a subsequent decrease in biofilm formation (7). The Csu downregulation caused by TMP and SMX, which act synergistically to inhibit synthesis of folate pathway intermediates and cause folate stress, can be overcome by the addition of the essential intermediate tetrahydrofolate (7). Csu belongs to the chaperone-usher pathway (CUP) family of pili and mediates bacterial adherence to abiotic surfaces, such as polystyrene and other materials that are commonly used in medical equipment (8–10). Csu repression in response to TMP/SMX-induced folate stress occurs in multiple A. baumannii strains; however, the mechanism underlying this response is unknown. In addition to Csu, we found that TMP/SMX also increases the expression of the *paa* operon (11). The *paa* operon is comprised of 13 genes (*paaABCDEFGHJKXYI*) encoding the enzymes necessary to degrade phenylacetic acid (PAA), an intermediate in the catabolism of the amino acid l-phenylalanine. PAA catabolism involves several steps, including the formation of PAA-coenzyme A (PAA-CoA) followed by a unique epoxidation step, which is dependent on PaaABCDE. Ultimately, PAA catabolism leads to the production of succinyl-CoA and acetyl-CoA, which then feed into the tricarboxylic acid (TCA) cycle under aerobic conditions (12, 13). Multiple transcriptomic studies performed with various A. baumannii strains have indicated that the *paa* operon is one of the most differentially regulated pathways in response to various environmental conditions, such as pleural fluid, imipenem, and blue light (14–18). Furthermore, PAA catabolic genes are highly expressed in cephalosporin-induced persister cells compared to planktonically grown bacteria and are differentially regulated between motile and nonmotile populations (16, 19). Although metabolic pathways are often regulated in response to environmental changes, the PAA pathway is the only amino acid catabolic pathway in Acinetobacter that is so consistently and highly regulated under many diverse conditions.

Importantly, studies have implicated the *paa* operon in the pathogenesis of multiple Gram-negative bacteria, including Burkholderia cenocepacia and A. baumannii (20–23). For example, inactivation of the upper PAA catabolic pathway (*paaABCDE*) leads to decreased virulence of B. cenocepacia in a Caenorhabditis elegans model (20, 23). In A. baumannii, the *paa* operon is controlled by the virulence-associated two-component system (TCS) GacSA and has been implicated in pathogenesis in murine septicemia and zebrafish infection models (21, 22). It was proposed that the diminished virulence of the Δ*paaA* strain in zebrafish was due to increased levels of secreted PAA, which acted as a neutrophil chemoattractant (21). However, the effect of PAA on the physiology of A. baumannii, especially in modern clinical isolates, is still not understood.

PAA has known signaling properties in plant cells, where it acts as an auxin to regulate plant growth and development (24, 25). In addition to their roles in plant development, plant-produced auxins are known to have regulatory effects on the surrounding soil bacteria (24, 26). For example, Pseudomonas putida senses PAA using the Aer2 receptor, swimming toward PAA to utilize it as a carbon source (27). Furthermore, endogenously produced PAA inhibits expression of the type III secretion system (T3SS) in Pseudomonas aeruginosa in a growth-phase-dependent manner (28). This piece of data suggests that PAA may serve as an important signaling molecule in multiple bacteria; however, little is known about its importance in Acinetobacter. In this work, we report a novel role for PAA as a mediator of the stress response of A. baumannii.

## RESULTS

### Phenylacetic acid degradation is differentially regulated under antibiotic stress.

**FIG 1:**
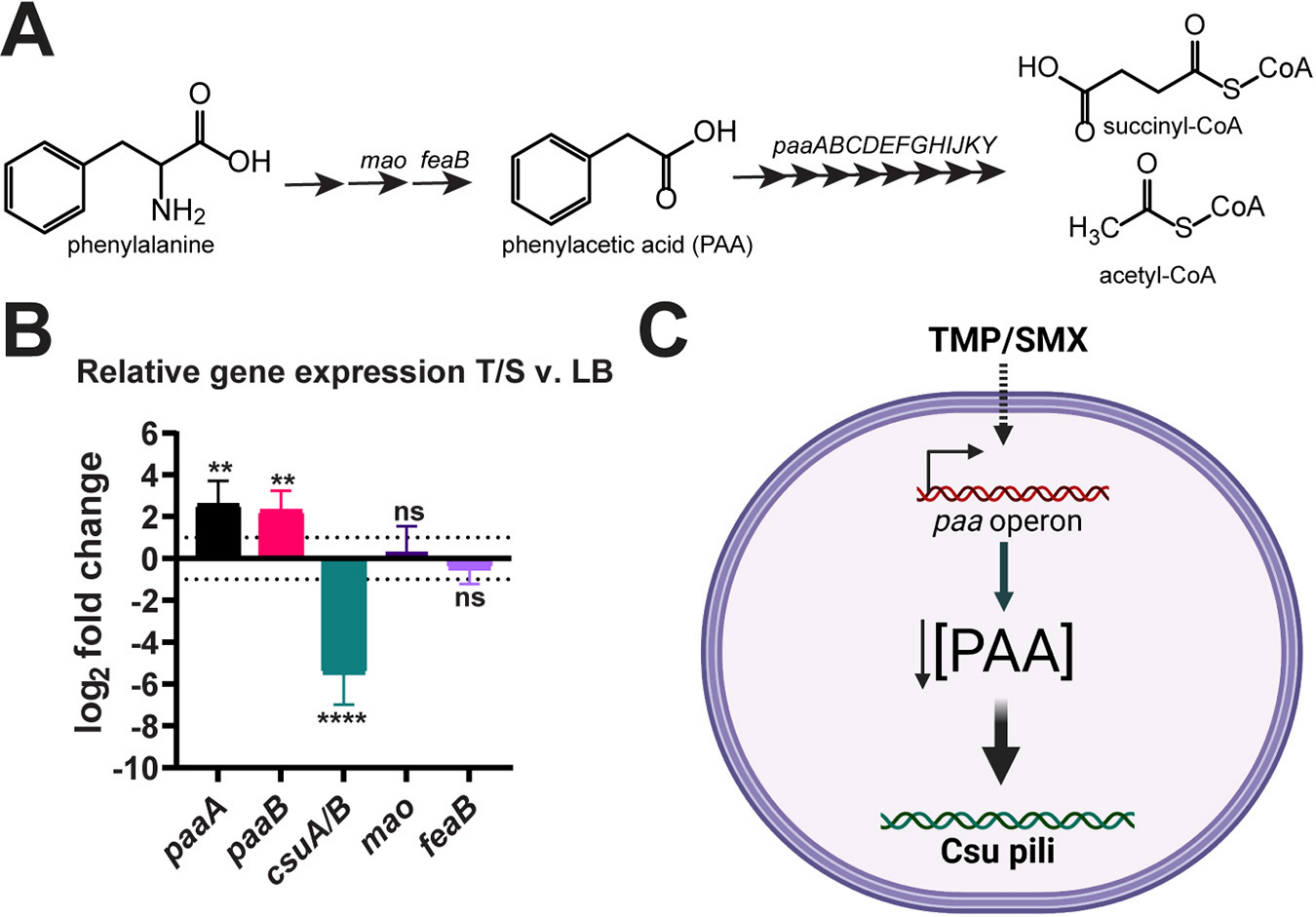
The *paa* operon is upregulated under TMP/SMX treatment. (A) Schematic of phenylalanine and PAA catabolism, where *mao* encodes the monoamine oxygenase, *feaB* encodes the phenylacetaldehyde dehydrogenase, and the *paa* operon (*paaABCDEFGHIJKY*) encodes enzymes responsible for PAA’s degradation to succinyl- and acetyl-CoA. (B) Relative gene expression of *csuA*/*B* major pilin and PAA metabolism genes in WT Ab17978 grown in TMP/SMX compared to Ab17978 grown in LB, as determined by qRT-PCR. Results are depicted as the mean ± standard deviation (SD) from at least 3 biological replicates and are depicted as log_2_ fold change. Statistical analyses were performed on Δ*C_T_* values using analysis of variance (ANOVA) and Tukey’s multiple-comparison test (*, *P* < 0.05; **, *P* < 0.01). (C) Proposed model of PAA regulation in A. baumannii. Created with BioRender.com.

Previously, we observed via transcriptomic analysis that Ab17978 responds to TMP/SMX treatment by upregulating the *paa* operon, which encodes the enzymes involved in phenylalanine catabolism, specifically the steps downstream of the intermediate PAA (7). Two enzymes involved in the conversion of phenylalanine to PAA are the monoamine oxidase encoded by *mao* and the phenylacetaldehyde dehydrogenase encoded by *feaB* (Fig. 1A) (29). Neither *mao* nor *feaB* was significantly upregulated during TMP/SMX treatment according to RNA sequencing (RNA-Seq), suggesting that this response is not simply an increase in phenylalanine metabolism (7). To confirm our previous transcriptomic analyses, we performed reverse transcription-quantitative PCR (RT-qPCR) and determined that *paaA* and *paaB* levels are increased approximately 7-fold at 2 h post-TMP/SMX treatment compared to Ab17978 grown in LB (Fig. 1B). In contrast, levels of expression of *mao* and *feaB* were not significantly altered under these conditions. The observed increase in *paa* operon expression was coupled with an ~15-fold decrease in expression levels of *csuA*/*B*, which encodes the CsuA/B major pilin subunit of Csu pili (Fig. 1B). This is consistent with our previous work demonstrating repression of Csu pilus expression under TMP/SMX treatment (7). These results prompted us to hypothesize that environmental signals, such as antibiotic treatment, cause an increase in the expression of the *paa* operon, impacting PAA levels in the cell and leading to downstream responses, such as the repression of Csu pili (Fig. 1C).

### PAA levels regulate biofilm formation and Csu expression.

**FIG 2:**
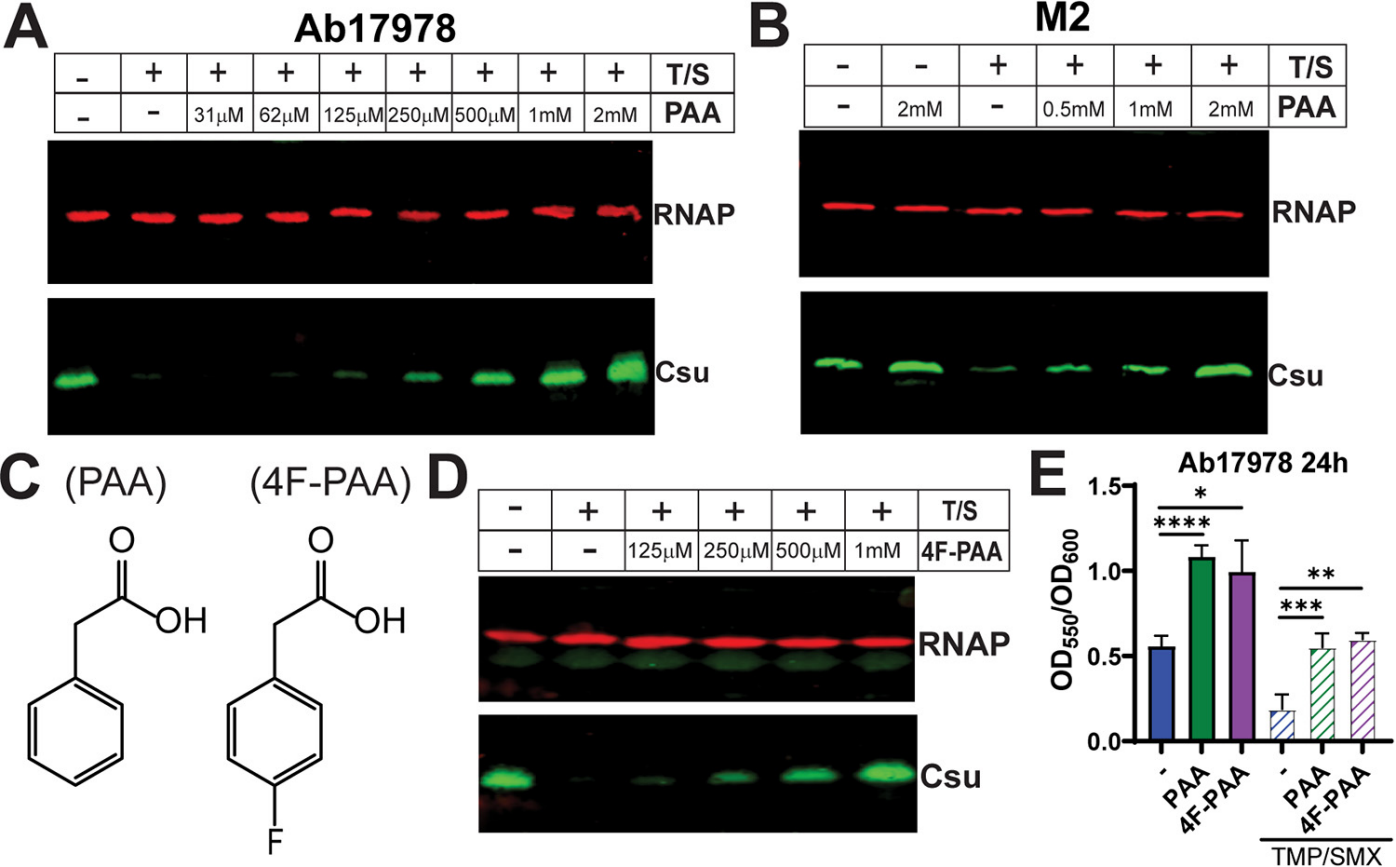
PAA and 4F-PAA induce Csu pilus production under TMP/SMX treatment in multiple Acinetobacter strains. (A) Western blot measuring expression of Csu pili in OD-normalized whole cells of Ab17978. WT Ab17978 was grown for 4 h in LB with or without TMP/SMX (T/S) at 3 μg/mL and 15 μg/mL, respectively. TMP/SMX-treated cells were also grown with increasing amounts of PAA. RNA polymerase (RNAP) was used as a loading control, and Csu was probed with an anti-CsuA/B antibody targeting the major Csu pilin. (B) Western blot analysis of Csu expression in whole cells of A. nosocomialis M2. Cultures were grown similarly to Ab17978, except T/S concentrations were at 1 μg/mL and 4 μg/mL, respectively. (C) Molecular structures of PAA and the fluorinated PAA derivative 4-fluorophenylacetic acid (4F-PAA). (D) Csu Western blot of Ab17978 whole cells grown in LB, T/S, or T/S with increasing concentrations of 4F-PAA. All Western blots shown are representative of at least three independent experiments. (E) Biofilm formation of Ab17978 grown in the presence of 2 mM PAA or 4F-PAA in the presence or absence of TMP/SMX. Biofilm was measured via crystal violet staining in 96-well plates after 24 h of static growth. OD_550_ measurements of crystal violet were normalized to OD_600_ values, and the results are represented as the mean ± SD of the OD_550_/OD_600_ ratios. Biofilms are representative of at least 3 independent experiments, and statistical analysis was done via *t* test with Welch’s correction (*, *P < *0.05; **, *P < *0.01; ***, *P < *0.005; ****, *P < *0.001).

We confirmed, by Western blotting using an antibody for the CsuA/B major pilin, that TMP/SMX treatment almost completely abolished Csu expression in Ab17978, consistent with previously published results (Fig. 2A) (7). We found that the TMP/SMX-mediated effect on Csu expression can be overcome by the addition of exogenous PAA in a concentration-dependent manner, reaching expression levels comparable to that of the LB control at 0.5 mM PAA (Fig. 2A). Similar results were obtained in Acinetobacter nosocomialis M2, a strain of a medically relevant species, showing that this phenotype is not specific to Ab17978 (Fig. 2B). Moreover, addition of increasing concentrations of fluoro-phenylacetic acid (4F-PAA), a 4-fluorinated-derivative of PAA (Fig. 2C), also restored CsuA/B expression under TMP/SMX treatment (Fig. 2D). Both PAA and 4F-PAA restore Csu expression to LB-like levels at 0.5 mM (see Fig. S1A in the supplemental material), and growth of Ab17978 is not inhibited under these conditions, suggesting that these molecules are not toxic at these concentrations (Fig. S1B). Ab17978 can grow in M9 minimal medium supplemented with PAA, but not 4F-PAA, as the only carbon source, indicating that 4F-PAA is not metabolized into TCA cycle intermediates (Fig. S1C). These results indicate that restoration of Csu levels by PAA was not due to PAA being used as an additional carbon source. Csu pili are one of the main determinants of biofilm formation in Ab17978 (8, 9). We found that, in agreement with Csu Western blot data, exogenously added PAA or 4F-PAA induced biofilm formation in Ab17978, as measured by crystal violet binding (Fig. 2E). Biofilm formation in a Δ*csuD* mutant was completely abolished in the presence and absence of exogenous PAA, confirming that increased biofilm formation in response to PAA is dependent on Csu expression (see Fig. S2 in the supplemental material). Together, these results implicate PAA in a putative signaling pathway induced by antibiotic stress and resulting in physiological changes (e.g., Csu expression).

10.1128/mbio.01863-21.1 FIG S1 Physiological responses of Ab17978 to PAA or 4F-PAA. (A) Quantification of Csu expression of Ab17978 grown with TMP/SMX (T/S) and 0.5 mM of either PAA or 4F-PAA. Each point represents one biological replicate, with error bars representing the SD. To quantify, Csu intensity was normalized to RNAP intensity, and these values were normalized to the LB control, which was set at 1. Statistical analyses were performed by the Mann-Whitney *U* test (*, *P < *0.05). (B) Growth curve of Ab17978 in T/S with 0.5 mM PAA or 4F-PAA suspended in ethanol, the same concentrations used for Csu Western blots. Ethanol was used as a solvent control. (C) Growth curve of WT Ab17978 in M9 minimal medium supplemented with 5 mM PAA or 4F-PAA suspended in DMSO. DMSO alone acts as a carbon-negative control. Download FIG S1, TIF file, 0.4 MB. Copyright © 2022 Hooppaw et al. 2022 Hooppaw et al. https://creativecommons.org/licenses/by/4.0/ This content is distributed under the terms of the Creative Commons Attribution 4.0 International license.

10.1128/mbio.01863-21.2 FIG S2 PAA-mediated increase in biofilm formation is Csu dependent. Biofilm formation of the WT Ab17978 strain and a Δ*csuD* mutant was assessed via crystal violet staining after 24 h of static growth in LB or LB with PAA, TMP/SMX, or PAA+TMP/SMX. Results are representative of 3 biological replicates. Download FIG S2, TIF file, 1.2 MB. Copyright © 2022 Hooppaw et al. 2022 Hooppaw et al. https://creativecommons.org/licenses/by/4.0/ This content is distributed under the terms of the Creative Commons Attribution 4.0 International license.

### PAA induces significant proteomic changes.

**FIG 3:**
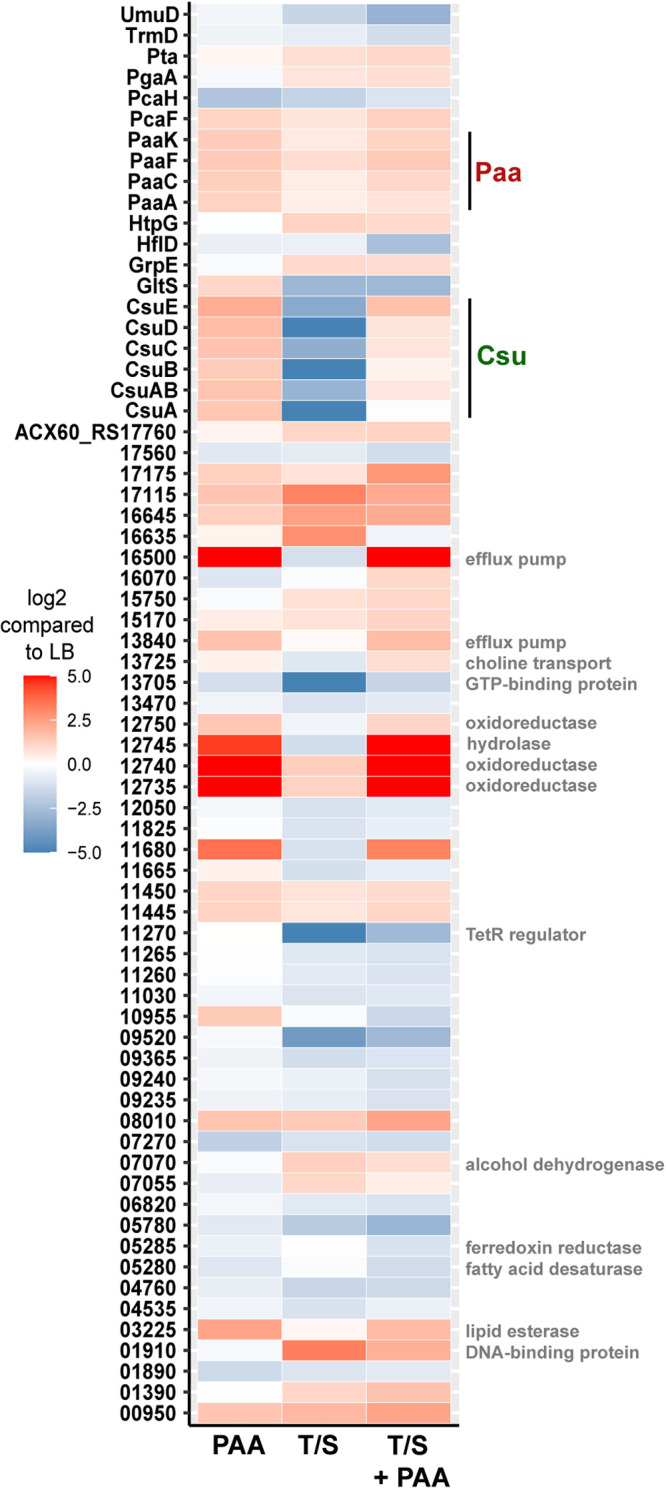
PAA induces global changes in protein expression. Heat map of proteins detected by mass spectrometry to be differentially expressed in Ab17978 whole cells treated with PAA, TMP/SMX (T/S), or TMP/SMX+PAA compared to the LB control. To prepare protein samples, Ab17978 was grown for 4 h under each of the 4 conditions before being pelleted, lysed, and acetone precipitated. TMP and SMX were used at 3 μg/mL and 15 μg/mL, respectively, and PAA was used at 0.5 mM. Paa proteins are highlighted in red, and Csu is highlighted in green. Other proteins were labeled in gray if they were (i) divergently regulated between TMP/SMX and TMP/SMX+PAA conditions and (ii) had putative annotated functions. Each colored block represents the mean of 4 biological replicates. Student *t* tests were used to compare groups, with a cutoff *P* value of <0.05.

Our results show that Ab17978 modulates expression of Csu pili in a PAA-dependent fashion. To identify other proteins that are differentially regulated in response to PAA, we performed quantitative differential proteomics. Wild-type (WT) Ab17978 was grown in LB, LB+TMP/SMX, LB+PAA, or LB+TMP/SMX+PAA for 4 h before whole cells were harvested and analyzed via tandem mass spectrometry (MS/MS). In agreement with our previous results, proteomic analysis showed that levels of expression of proteins involved in the biogenesis of Csu pili were significantly downregulated in TMP/SMX-treated cells, while levels of expression of proteins involved in PAA degradation were significantly upregulated. Addition of PAA overcame the effect of TMP/SMX on Csu proteins (Fig. 3). Several other proteins followed a similar pattern of expression, where addition of PAA reverted or overcame the effects of TMP/SMX treatment (see Table S1 in the supplemental material). The most prominent of these are 4 proteins encoded together in an operon that are strongly induced by PAA (ACX60_RS12735 to ACX60_RS12750). These proteins have putative hydrolase and oxidoreductase activities; however, their functions remain uncharacterized. Additional proteins include those with putative efflux pump activities (ACX60_RS16500 and ACX60_RS13840), a putative GTP-binding protein (ACX60_RS13705), and a TetR family regulator (ACX60_RS11270). Although further work is necessary to characterize the roles of these proteins in the PAA-induced response of A. baumannii, our results suggest that Csu downregulation is one of several responses to folate stress that can be reversed by PAA.

10.1128/mbio.01863-21.5 TABLE S1 Differential proteomics of Ab17978 in TMP/SMX versus TMP/SMX and PAA. Download Table S1, XLSX file, 0.02 MB. Copyright © 2022 Hooppaw et al. 2022 Hooppaw et al. https://creativecommons.org/licenses/by/4.0/ This content is distributed under the terms of the Creative Commons Attribution 4.0 International license.

### Mutagenesis of *paaB* alters the expression of multiple genes in the presence of antibiotics.

**FIG 4:**
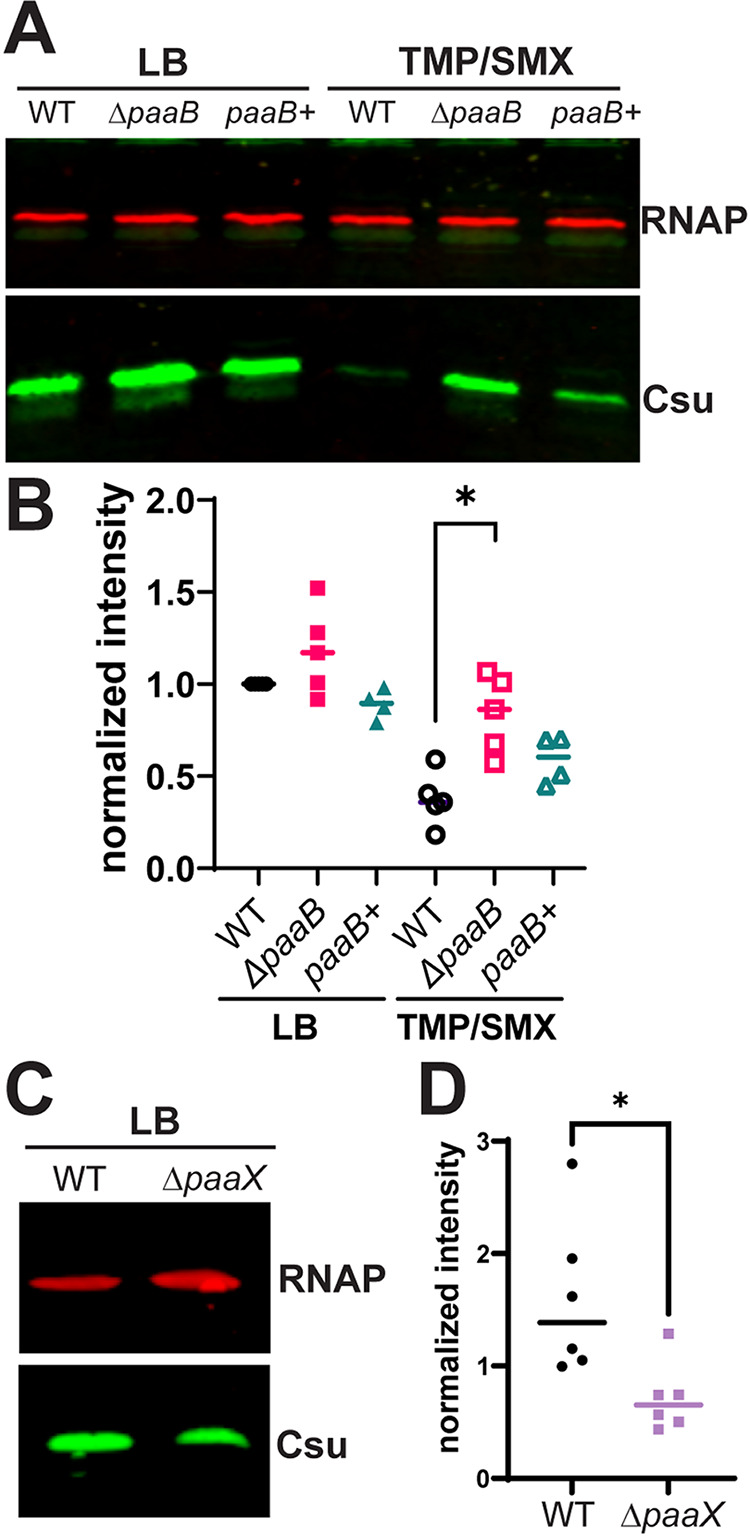
PAA degradation is important for the response to TMP/SMX. (A) Csu expression of Ab17978 WT, Δ*paaB*, and *paaB+* strains via anti-CsuA/B Western blot. The three strains were grown in LB or LB+TMP/SMX (3/15 μg/mL) for 4 h before whole cells were harvested and separated by SDS-PAGE. (B) Normalized intensity and quantification of panel A. Csu/RNAP intensity ratios were normalized to Csu/RNAP values in the WT strain grown in LB. (C) Csu expression of the WT and Δ*paaX* strains when grown in LB. (D) Quantification of panel C. The intensity of Csu was normalized to RNAP. Points represent independent biological replicates, and significance was determined via Mann-Whitney *U* test (*, *P < *0.05).

Our experiments show that exogenous addition of PAA to A. baumannii cultures increases Csu expression, reversing the repression of Csu caused by TMP/SMX. To enable our investigation into the role of the PAA pathway *in vivo*, we sought to manipulate PAA levels by generating a *paaB* mutant in Ab17978. *paaB* is part of the multicomponent monooxygenase complex that is involved in one of the first steps of PAA catabolism (13, 21). Previous work in Burkholderia cenocepacia has shown that *paaB* is essential for PAA catabolism, and *paaB* mutants accumulate PAA (23). We confirmed that the Ab17978 Δ*paaB* mutant grows similarly to the WT in LB (see Fig. S3A in the supplemental material) but is unable to grow on PAA as the sole carbon source, and in *trans* complementation of *paaB* (*paaB*+) restored growth on PAA (Fig. S3B). As predicted, the accumulation of PAA in the Δ*paaB* strain mimicked the effect of exogenous addition of PAA, leading to increased biofilm formation (Fig. S3C). WT, Δ*paaB*, and *paaB*+ strains grown in LB exhibited comparable levels of Csu, as determined by Western blotting (Fig. 4A). However, levels of Csu in the Δ*paaB* strain were considerably higher than in the WT strain when cells were grown in TMP/SMX (Fig. 4A). The *paaB+* complemented strain was able to partially repress Csu expression, as the normalized Csu intensity of this strain was not significantly different from that of the WT (Fig. 4B). We next investigated how overexpression of the *paa* operon affects Csu expression. Thus, we generated a Δ*paaX* mutant in Ab17978. PaaX is known to be the main regulator of the *paa* operon in Escherichia coli, where it was characterized to be a repressor (30). Csu expression in the Δ*paaX* mutant was approximately 50% of that in the WT strain, further supporting the hypothesis that reduced PAA levels are partially responsible for repressed Csu expression in Ab17978 (Fig. 4C and D). Together, these data further support our hypothesis that PAA catabolism is important for the response of Ab17978 to folate stress.

10.1128/mbio.01863-21.3 FIG S3 Characterization of growth and biofilm formation of the Ab17978 *paaB* mutant. (A) Growth curve of WT, Δ*paaB*, and *paaB+* strains in (A) LB or (B) M9 minimal medium supplemented with 5 mM PAA as the sole carbon source. Cultures were diluted to an OD_600_ of 0.01 and grown at 37°C with shaking. (C) Biofilm formation of WT, Δ*paaB*, and *paaB+* strains in LB after 24 h of static growth. Statistical analysis was performed via *t* test with Welch’s correction (**, *P < *0.01; ***, *P < *0.005). All results are representative of 3 biological replicates. Download FIG S3, TIF file, 0.6 MB. Copyright © 2022 Hooppaw et al. 2022 Hooppaw et al. https://creativecommons.org/licenses/by/4.0/ This content is distributed under the terms of the Creative Commons Attribution 4.0 International license.

Proteomic changes may not necessarily reflect changes in gene expression, so we performed transcriptomic experiments on whole cells of the Ab17978 WT and Δ*paaB* mutant strains grown in LB or LB+TMP/SMX (see Table S2 in the supplemental material). We found that, in the absence of antibiotic stress, 19 genes were significantly upregulated in the Δ*paaB* strain relative to the WT. In agreement with our proteomic data, these included the 13 genes present in the *paa* operon (Table S2). Induction of the *paa* operon is consistent with a metabolic response to the accumulation of PAA, which occurs in Δ*paaB* strains (21, 23). Additionally, *adeIJK* genes, which encode an RND family efflux pump, were also increased in the *paaB* mutant. The AdeIJK efflux pump of A. baumannii is thought to be a broad-spectrum pump with a preference for amphiphilic compounds (31). Previously, both *csu* and *paa* genes were shown to be downregulated in a Δ*adeIJK* mutant of strain AB5075 (32). This suggests a regulatory connection between these pathways that is conserved between A. baumannii strains. Future work is needed to understand the exact mechanisms behind the cross talk between the PAA pathway, Csu pili, and efflux pumps in Acinetobacter. Several genes related to CoA metabolism were also downregulated in the *paaB* mutant compared to WT (ACX60_RS09235, ACX60_RS09240, and ACX60_RS06820). This is consistent with our proteomic data which shows these proteins downregulated in response to exogenous PAA addition (Fig. 3; Table S1). Contrasting the changes seen in LB, the WT and Δ*paaB* mutant displayed a broader repertoire of differentially expressed genes in the presence of folate stress. In agreement with our model, the *csu* operon was more highly expressed in the Δ*paaB* strain. Remarkably, many other genes were differentially regulated in the presence of the antibiotics (see Table S3 in the supplemental material). Although the function of most of these genes needs to be experimentally determined, we propose that genes that are differentially expressed in the WT and *paaB* mutant constitute the “PAA-dependent regulon.”

10.1128/mbio.01863-21.6 TABLE S2 Differentially regulated chromosomal genes in the Δ*paaB* versus WT strain in LB. Download Table S2, PDF file, 0.1 MB. Copyright © 2022 Hooppaw et al. 2022 Hooppaw et al. https://creativecommons.org/licenses/by/4.0/ This content is distributed under the terms of the Creative Commons Attribution 4.0 International license.

10.1128/mbio.01863-21.7 TABLE S3 Differentially regulated chromosomal genes in the Δ*paaB* versus WT strain in TMP/SMX. Download Table S3, PDF file, 0.1 MB. Copyright © 2022 Hooppaw et al. 2022 Hooppaw et al. https://creativecommons.org/licenses/by/4.0/ This content is distributed under the terms of the Creative Commons Attribution 4.0 International license.

### PAA catabolism is important for growth in antibiotics.

**FIG 5:**
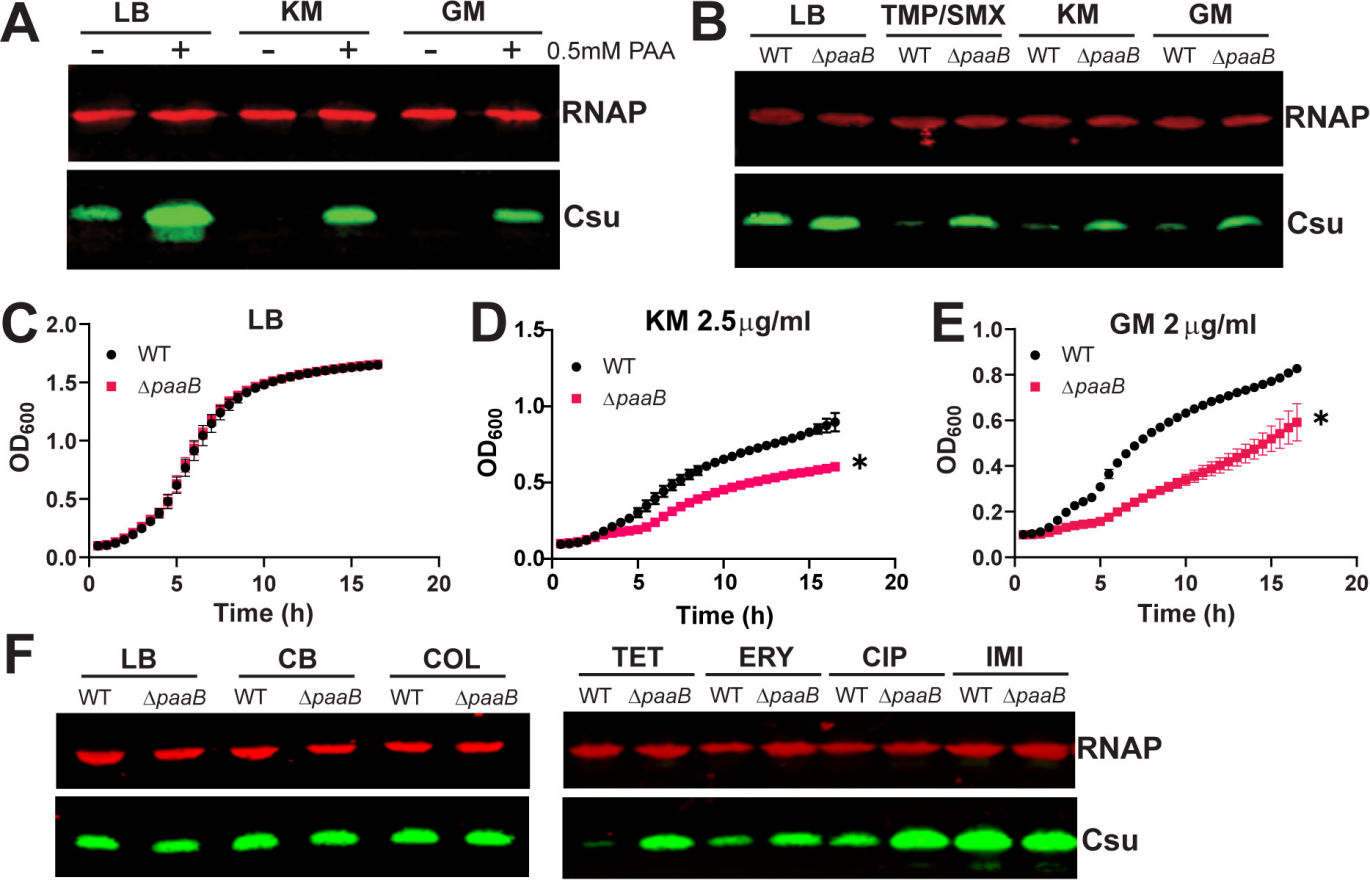
PAA interrupts the antibiotic response of Ab17978. (A) Csu Western blot of Ab17978 grown in LB with kanamycin (1.5 μg/mL) or gentamicin (1.75 μg/mL), with and without 0.5 mM PAA. (B) Csu expression of WT Ab17978 and Δ*paaB* grown in LB, TMP/SMX, kanamycin, or gentamicin. (C to E) Representative growth curves of the WT and Δ*paaB* strains in LB, kanamycin (2.5 μg/mL), or gentamicin (2 μg/mL). Cultures were normalized to 0.01 and grown for 16 h with shaking at 37°C. Asterisks indicate significance based on multiple unpaired *t* tests at each time point. Significance of antibiotic growth curves was achieved starting at the 6th time point as determined by Student's *t* test (*P < *0.01). (F) Csu expression of WT Ab17978 and Δ*paaB* strains grown in different antibiotics. CB, carbenicillin (64 μg/mL); CIP, ciprofloxacin (0.125 μg/mL); COL, colistin (0.5 μg/mL); ERY, erythromycin (4 μg/mL); IMI, imipenem (0.125 μg/mL); TET, tetracycline (0.125 μg/mL). All results are representative of at least 3 independent experiments.

In the previous experiments, we employed TMP/SMX to induce antibiotic stress. TMP/SMX is a cocktail of drugs that synergistically inhibits synthesis of tetrahydrofolate (THF), which is essential for nucleotide synthesis, leading to folate stress (7, 33). However, PAA could be part of a general response to antibiotic stress rather than being specific to folate stress. Supporting this concept, we found that Csu expression was repressed in Ab17978 grown in the presence of either kanamycin or gentamicin at a concentration that was half of the MIC. Exogenous addition of 0.5 mM PAA overruled Csu repression (Fig. 5A). Similarly, the Δ*paaB* mutant strain grown in sub-MICs of these aminoglycosides was unable to repress Csu expression (Fig. 5B). Importantly, sub-MICs of kanamycin or gentamicin caused a significant inhibition of growth in the *paaB* mutant compared to the WT strain, indicating that the *paaB* mutant strain cannot properly respond to aminoglycoside-induced stress (Fig. 5C to E). To understand if this PAA-dependent response is limited to aminoglycosides and TMP/SMX, we performed Csu Western blots on WT and Δ*paaB* cultures grown in sub-MICs of diverse antibiotics. We found that antibiotics targeting the bacterial cell wall (carbenicillin and imipenem) or outer membrane (colistin) did not alter Csu expression in either strain (Fig. 5F) In contrast, antibiotics targeting DNA replication (ciprofloxacin) or protein synthesis (erythromycin and tetracycline) repressed Csu in the WT strain, but not the Δ*paaB* mutant (Fig. 5F). Our results indicate that PAA can mediate the response of A. baumannii to stress induced by multiple classes of antibiotics, specifically those with cytoplasmic targets. It has also been shown that MumR, a transcriptional regulator involved in manganese uptake and hydrogen peroxide (H_2_O_2_) tolerance, influences the expression of the *paa* operon, and an Ab17978 mutant in the *paa* operon displayed increased sensitivity to H_2_O_2_ and oxidative stress (34). Together with our observations, this suggests that PAA may play a more general role in responding to different types of stressors.

### PAA degradation is important for stress tolerance and virulence of the uropathogenic strain UPAB1.

**FIG 6:**
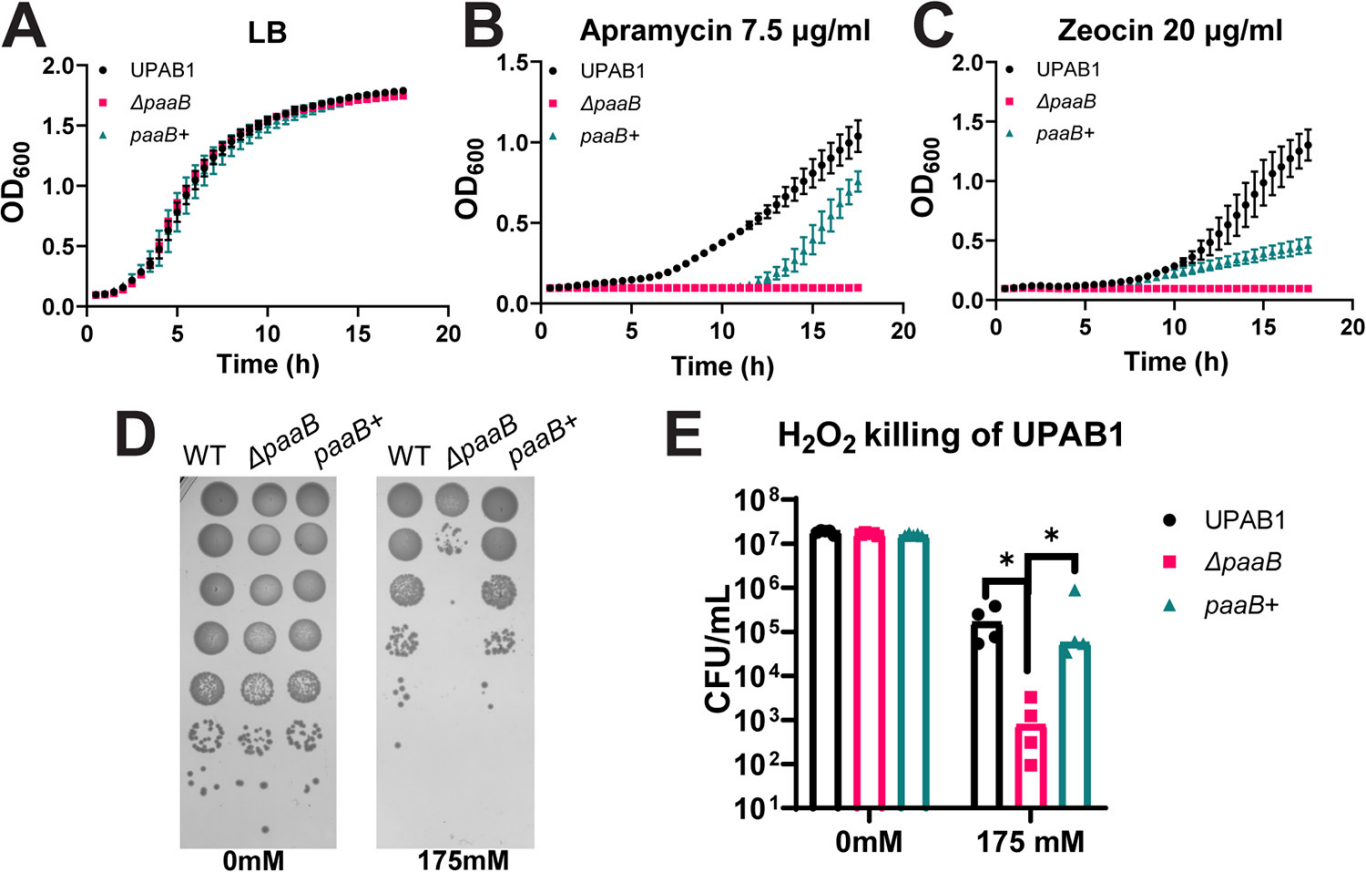
PAA catabolism is important for stress tolerance in the uropathogenic strain, UPAB1. (A to C) Representative growth curves of UPAB1 WT, Δ*paaB*, and *paaB+* strains. (D) Representative H_2_O_2_ killing of UPAB1 strains. The WT, Δ*paaB*, and *paaB+* strains were grown in LB to mid-logarithmic phase before being treated with 0 mM or 175 mM H_2_O_2_ for 30 min. Serial dilutions were spotted to measure survival. (E) Quantification of H_2_O_2_ killing of UPAB1 strains shown in recoverable CFU/mL. Points represent technical replicates from at least 3 biological replicates, with bars representing the median. Statistical analysis using the Mann-Whitney *U* test was performed (*, *P < *0.05).

Although Ab17978 is a well-studied model strain used to study Acinetobacter physiology, it lacks many of the virulence factors and antibiotic resistance cassettes that are present in recent clinical isolates of A. baumannii (35, 36). To investigate antibiotic sensitivity in a modern strain, we generated a Δ*paaB* mutant in the recent clinical A. baumannii isolate UPAB1, a urinary isolate that is virulent in multiple mouse models (37). As seen in Ab17978, UPAB1 Δ*paaB* does not exhibit any growth defects in LB compared to WT (see Fig. S4A in the supplemental material), but the Δ*paaB* strain is not able to grow on M9 medium with PAA as the sole carbon source. Importantly, chromosomal insertion of *paaB* (*paaB+*) complemented this phenotype (Fig. S4B). Similar to Ab17978 Δ*paaB*, UPAB1 Δ*paaB* also formed higher levels of biofilm compared to the WT and *paaB*+ strains, demonstrating that PAA catabolism plays an important role in biofilm formation in multiple Acinetobacter strains (Fig. S4C). UPAB1 is highly resistant to many antibiotics, including kanamycin and gentamicin at high concentrations (MIC of >400 μg/mL). Therefore, we used two antibiotics to which UPAB1 is not resistant, the aminoglycoside apramycin and the glycopeptide zeocin. We found that the *paaB* mutant displayed no growth under both apramycin and zeocin treatments compared to the WT and *paaB+* strains, while all three strains grew similarly in LB (Fig. 6A to C). This prompted us to measure the antimicrobial susceptibility profiles of WT and *paaB* mutants in UPAB1 and Ab17978. Indeed, we found that the MIC of apramycin is 4 times lower in UPAB1 Δ*paaB* than WT UPAB1, consistent with previous growth curves (Table 1). Furthermore, modest decreases in MIC were observed in *paaB* mutants for ciprofloxacin, erythromycin, and zeocin in both UPAB1 and Ab17978 (Table 1). This piece of data further supports our hypothesis that a PAA pathway-mediated antibiotic stress response is conserved among A. baumannii strains and that interfering with this response can decrease antibiotic resistance in a clinical isolate.

**TABLE 1:** Susceptibility of *paaB* mutants to antibiotics^*a*^

Antibiotic	MIC (μg/mL) for:			
	Ab17978		UPAB1	
	WT	Δ*paaB*	WT	Δ*paaB*
Colistin	1	1	2	2
Ciprofloxacin	**1**	**0.5**	**32**	**16**
Chloramphenicol	64	64	64	64
Erythromycin	**32**	**16**	**16**	**8**
Gentamicin	4	4	>512	>512
Imipenem	0.5	0.5	32	32
Kanamycin	4	4	**>512**	**256**
Tetracycline	1	1	16	16
Zeocin	**64**	**32**	**32**	**16**
Apramycin	8	8	**32**	**8**

a MIC values that differ between WT and *paaB* mutants are bolded.

10.1128/mbio.01863-21.4 FIG S4 Characterization of growth and biofilm formation of the UPAB1 *paaB* mutant. (A) Growth curve of WT, Δ*paaB*, and *paaB+* strains in (A) LB or (B) M9 minimal medium supplemented with 5 mM PAA as the sole carbon source. Cultures were diluted to an OD_600_ of 0.01 and grown at 37°C with shaking. (C) Biofilm formation of WT, Δ*paaB*, and *paaB+* strains in LB after 24 h of static growth. Statistical analysis was performed via *t* test with Welch’s correction (***, *P < *0.005; ****, *P < *0.001). All results are representative of 3 biological replicates. Download FIG S4, TIF file, 0.6 MB. Copyright © 2022 Hooppaw et al. 2022 Hooppaw et al. https://creativecommons.org/licenses/by/4.0/ This content is distributed under the terms of the Creative Commons Attribution 4.0 International license.

**FIG 7:**
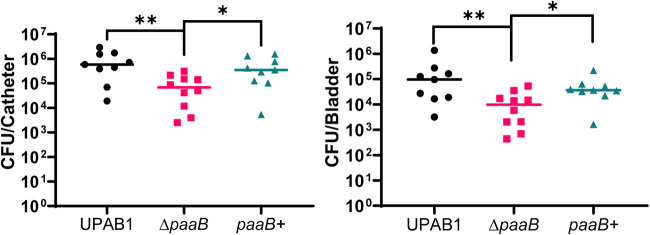
PAA catabolism is crucial for virulence in a CAUTI model. Catheter-implanted mice were infected with ~2 × 10^8^ CFU of the indicated strains. Following 24 h of infection, total numbers of CFU recovered were determined for the implants (left panel) and bladders (right panel). Each symbol represents an individual mouse. For each strain, the median value is shown as the horizontal line. Statistical analyses were performed using the Mann-Whitney *U* test (*, *P < *0.05; **, *P < *0.01).

As mentioned previously, an Ab17978 mutant in the *paa* operon displayed increased sensitivity to H_2_O_2_ (38). In agreement, we found that survival of UPAB1 Δ*paaB* was reduced by 2 logs compared to the WT strain after treatment with H_2_O_2_ (Fig. 6D and E). These results further support the notion that PAA is also involved in the response of A. baumannii to oxidative stress. Thus, we hypothesized that the inability of the *paaB* mutant to properly respond to this kind of stress may impact the virulence of UPAB1. While systemic models can serve as a useful benchmark for changes in virulence, they do not adequately represent the clinical manifestations of Acinetobacter disease. Because many A. baumannii infections occur in immunocompromised hosts, who often have medical implants, we employed a previously established catheter-associated urinary tract infection (CAUTI) murine model of infection that utilizes UPAB1, a urinary isolate, as a model (37). Briefly, 6- to 8-week-old female C57BL/6 mice were infected with 2 × 10^8^ CFU after a small silicone catheter was placed in each mouse urethra, and organs were plated for CFU 24 h postinfection. We found that the *paaB* mutant showed a significant reduction in recoverable CFU from the catheters and the bladders of infected mice (Fig. 7). The *paaB+* complemented strain showed CFU burdens comparable to WT levels. This confirms that PAA degradation is a critical pathway for the virulence of a modern clinical isolate in a CAUTI model.

## DISCUSSION

**FIG 8:**
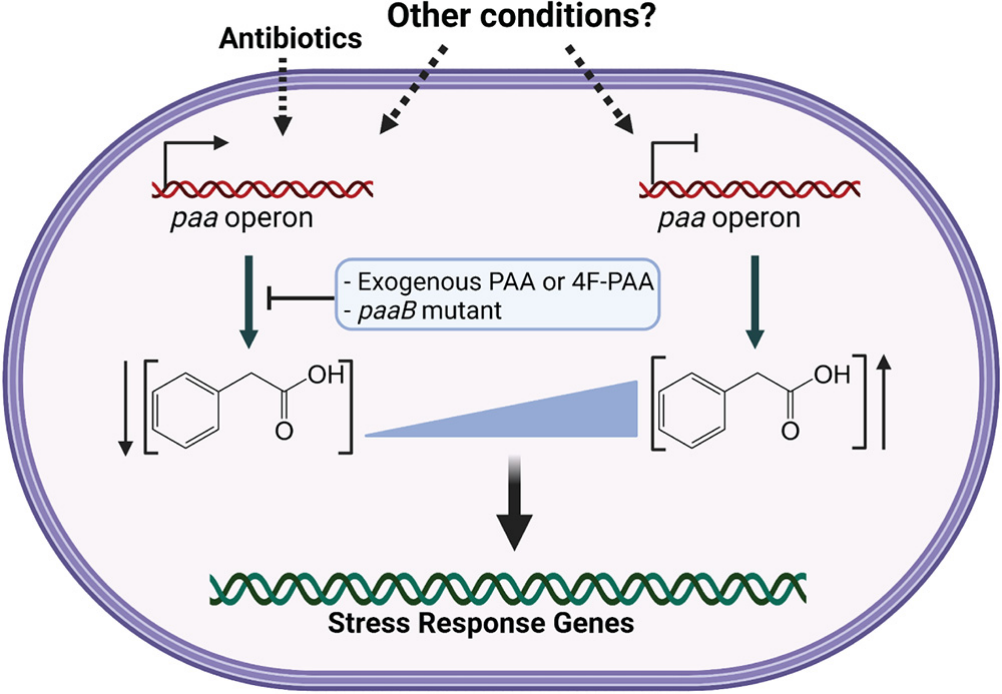
Proposed model of PAA signaling where antibiotic treatment leads to increased expression of the *paa* operon leading to decreased PAA levels, which ultimately regulates downstream responses such as Csu pili. Exogenous addition of PAA or 4F-PAA or the disruption of *paaB* can interfere with this response. We hypothesize that other conditions lead to decreased expression of the *paa* operon, meaning tight control of PAA levels is important for environmental adaptation of Acinetobacter.

A. baumannii is a threatening nosocomial pathogen that is highly resistant to antibiotics and tolerant to a wide range of stressors. Although the repertoire of virulence factors of A. baumannii remains largely uncharacterized, we know that it does not have the typical weapons like T3SS or T4SS that allow bacteria to interfere with the host immune response. In fact, Acinetobacter’s pathogenic potential is thought to rely on a “persist and resist” strategy that allows it to survive under a variety of harsh conditions (36). Therefore, investigating the mechanisms used by A. baumannii to adapt to stress will aid in our understanding of its pathogenesis and may lead to novel therapeutic discoveries. We and others have shown that the *paa* operon, which is responsible for PAA degradation, is one of the most differentially regulated pathways in response to numerous stress conditions and is strongly upregulated under antibiotic treatment (15, 16, 18, 39, 40). In this study, we show that the metabolite PAA interferes with the response of A. baumannii to various stressors. This activity is mimicked by its nonmetabolizable derivative 4F-PAA, which indicates this role is independent of PAA catabolism. We determined that increased concentrations of PAA, either through exogenous treatment or through mutation of the PAA degradation pathway, induce expression of Csu pili, overcoming antibiotic-mediated repression of Csu. Our results also confirmed that PaaX, the repressor of the *paa* operon, is involved in the regulation of Csu. Furthermore, we established that *paaB* mutant strains, which are known to accumulate PAA (21, 23), are sensitive to oxidative stress and exhibit reduced antibiotic tolerance and resistance. Finally, we demonstrated that a functional PAA catabolism pathway is critical for virulence of a clinical isolate in a murine CAUTI model. Taken together, this is the first evidence that PAA acts as a regulatory signal in multiple Acinetobacter strains. We propose a model in which environmental cues lead to an increase in *paa* operon expression, which controls cellular PAA levels and ultimately leads to changes in gene expression to control pilus expression, biofilm formation, and the response to antibiotics and oxidative stress Although folate stress and certain conditions increase *paa* expression (15–18, 39), reports have shown that *paa* expression is strongly repressed as a response to other environmental changes (14, 38). Therefore, it is tempting to speculate that cells respond to both high and low concentrations of PAA by regulating the expression of different gene subsets (Fig. 8).

It is not yet understood how the PAA signaling cascade fits into the global regulatory networks in A. baumannii. However, there are several regulatory proteins that have been shown to act upstream of the *paa* operon. We confirm that PaaX is important for regulation of Csu, which is consistent with overexpression of the *paa* operon. It is possible that PaaX interacts with other regulatory components to dynamically regulate *paa* operon expression under a variety of environmental conditions. In fact, the *paa* operon is also strongly regulated by the GacSA TCS in A. baumannii. GacS is an important virulence-associated histidine kinase that has roles in regulating biofilm formation and motility, both in A. baumannii and in Pseudomonas species (22, 41). Interestingly, a *gacS* mutant of A. baumannii showed up to 200-fold repression of *paa* genes compared to the WT, indicating that GacS is a major regulator of the *paa* operon and could be responsible for regulating *paa* genes in response to environmental changes (22). Additional regulators that act upstream of the *paa* operon include BlsA, a photoreceptor that negatively regulates expression of *paa* genes in response to blue light (14), and MumR, a manganese-responsive transcriptional regulator of the oxidative stress response in A. baumannii (34), further suggesting that PAA degradation plays a role in adaptation to a variety of environmental conditions.

We have shown that the *paa* operon has downstream effects on the expression of several factors, including Csu pili. It is well established that Csu expression is also regulated by the BfmRS TCS (42), a TCS involved in antibiotic tolerance, membrane homeostasis, and desiccation resistance (43, 44). While multiple factors likely affect Csu regulation, further work is necessary to determine if PAA interacts with components of the Bfm system and how PAA induces Csu expression. The involvement of multiple regulators in *paa* operon regulation supports our model that PAA is an important player in multiple signaling cascades.

Previous studies in the Gram-negative bacteria P. aeruginosa and B. cenocepacia, have shown that PAA might interact with the quorum-sensing machinery of these strains (23, 45). However, in B. cenocepacia, it was discovered that PAA is converted to PAA-CoA to interfere with quorum-sensing-mediated virulence of this strain (46). We cannot rule out that PAA-CoA, rather than PAA, is the signaling molecule that is responsible for the appreciable phenotypes in Acinetobacter. PAA could potentially be converted to PAA-CoA by the *paaK*-encoded PAA-CoA ligase before exerting its effects on the cell. However, for this to happen, *paaK* should be regulated independently from the rest of the *paa* operon to allow for dynamic control over PAA-CoA levels. Our transcriptomic and proteomic data indicate that this is not the case. Furthermore, PAA was shown to accumulate in a *paaABCDE* mutant (21). A metabolomic analysis would aid in the determination of the exact levels of the different metabolites in the PAA pathway under different environmental conditions.

Previous reports have linked PAA to virulence in the model strains Ab17978 and ATCC 19606 (21, 22). It was shown that PAA produced by A. baumannii is sensed by zebrafish neutrophils, which are attracted to the site of infection, leading to greater bacterial killing and improved host survival (21). Here, we show that inactivation of PAA catabolism attenuates UPAB1 virulence in a murine CAUTI model. We propose that attenuation is due to the reduced capacity of the Δ*paaB* strain to respond to the stresses the bacterium encounters during infection, which may include oxidative, osmotic, and other types of stress. More experiments are required to determine how host and bacterial factors contribute to the phenotype of *paa* mutants *in vivo* and how this plays a role in Acinetobacter pathogenesis.

We have observed for the first time that PAA is involved in a signaling pathway in A. baumannii. The biggest remaining question is how PAA mediates these physiological changes. PAA behaves as a signaling molecule in multiple organisms from plant to bacteria; however, little is understood about PAA-receptor interactions. In plants, PAA is a phytohormone that regulates many important processes involved in growth and development (25). In fact, PAA can interact with different auxin coreceptors from the transport inhibitor response1/auxin signaling F-box (TIR1/AFB) family, leading to changes in gene expression in plant cells (25). In bacteria, the only characterized PAA-receptor interaction is in the rhizobacterium P. putida, where PAA, mostly produced by plants, is sensed by the methyl-accepting chemotaxis receptor Aer2 (27). In this system, P. putida senses PAA gradients using Aer2 and swims toward PAA to use it as a carbon source. While this cascade is for metabolic purposes, it does suggest that bona fide PAA receptors may exist in other bacteria, such as A. baumannii, that sense and respond to endogenously produced PAA. Therefore, we hypothesize that PAA is an interspecies, cross-kingdom signaling molecule.

Understanding the molecular basis underlying the PAA-mediated response to stress could open new avenues for drug development and research. In fact, some PAA derivatives are already part of a group of FDA-approved nonsteroidal anti-inflammatory drugs, some of which have been used to treat bacterial infections in murine models (47, 48). The therapeutic potential of PAA or PAA derivatives in interfering with the stress response of drug-resistant bacteria is extremely relevant to the growing field of antimicrobial resistance, warranting further research into their potential effects on Acinetobacter stress tolerance and pathogenesis.

## MATERIALS AND METHODS

### Bacterial strains and growth conditions.

The bacterial strains used in this study are listed in Table S4 in the supplemental material. Unless otherwise noted, strains were grown in lysogeny broth (LB) liquid medium at 37°C with shaking (200 rpm). When necessary, strains were grown in kanamycin (30 μg/mL), kanamycin (7.5 μg/mL), zeocin (50 μg/mL), gentamicin (15 μg/mL), trimethoprim (6 μg/mL), and/or sulfamethoxazole (30 μg/mL). Overnight cultures used for phenotypic assays were always grown without antibiotics.

10.1128/mbio.01863-21.8 TABLE S4 Bacterial strains and plasmids used in this study. Download Table S4, PDF file, 0.1 MB. Copyright © 2022 Hooppaw et al. 2022 Hooppaw et al. https://creativecommons.org/licenses/by/4.0/ This content is distributed under the terms of the Creative Commons Attribution 4.0 International license.

### Construction of *paa* mutants and complemented strains.

The primers used in this study are listed in Table S5 in the supplemental material. The *paaB* mutant of UPAB1 was constructed as done previously (49). Briefly, an FLP recombination target (FRT) site-flanked zeocin resistance cassette was amplified from a variant of pKD4 with primers harboring 18 to 25 nucleotides of homology to the flanking regions of the target gene, *paaB*. Upstream and downstream flanking regions of *paaB* were also amplified, and the 3 fragments were assembled via overlap extension PCR to form linear DNA (50). For Ab17978, a kanamycin cassette was amplified with 150-bp oligonucleotide primers with flanking regions of *paaB* or *paaX* to form the linear PCR product. Linear PCR products were then electroporated into competent Ab17978 or UPAB1 carrying pAT04, which expresses the RecAB recombinase (49). Mutants were selected on zeocin (50 μg/mL) for UPAB1 or kanamycin (7.5 μg/mL) for Ab17978 and confirmed by PCR. To remove the antibiotic cassettes, mutants were transformed with the plasmid pAT03, which expresses the FLP recombinase. Clean mutants were confirmed by PCR and sequencing.

10.1128/mbio.01863-21.9 TABLE S5 Oligonucleotides used in this study. Download Table S5, PDF file, 0.09 MB. Copyright © 2022 Hooppaw et al. 2022 Hooppaw et al. https://creativecommons.org/licenses/by/4.0/ This content is distributed under the terms of the Creative Commons Attribution 4.0 International license.

Complementation of *paaB* mutants was done using a mini-Tn*7*-based, four-parental conjugation technique for chromosomal insertion that was previously described (51). Briefly, *paaABC* and 300 bp upstream of *paaA* were amplified from the Ab17978 chromosome and inserted into pUC18-miniTn7-Gm (51) using Hi-Fi DNA assembly mix (New England Biolabs) to make pUCT18-miniTn7-Gm-*paaABC.* For UPAB1, *paaABCDEFG* and 300 bp upstream of *paaA* were amplified from the chromosome of UPAB1 and inserted into pUCT18-miniTn7-Zeo (52). This vector was then linearized and religated to include only *paaAB* to create pUCT18-miniTn7-Zeo-*paaAB*. To create *paaB+* complemented strains, overnight cultures of either Ab17978 Δ*paaB* or UPAB1 Δ*paaB* recipients, HB101(pRK2013), EC100D(pTNS2) for UPAB1 or SM10(pTNS3) for Ab17978, and either TOP10(pUCT18-miniTn7-Zeo-*paaAB*) for UPAB1 or TOP10(pUCT18-miniTn7-Gm-*paaABC*), were normalized to an optical density at 600 nm (OD_600_) of 2.0, and 100 μL of each culture was added to 600 μL LB. The bacterial mixtures were washed 2 times with 1 mL LB, resuspended in 25 μL LB, spotted onto a low-salt Luria agar plate, and incubated overnight at 37°C. For UPAB1, resuspended bacteria were plated on Luria agar with chloramphenicol (12 μg/mL) to select against E. coli strains and zeocin (50 μg/mL) to select for mini-Tn*7* recipients. For Ab17978, Vogel-Bonner minimal medium with 0.2% glycerol, chloramphenicol (12 μg/mL), and gentamicin (100 μg/mL) was used for selection, similar to what was previously described (53). For UPAB1, the *paaB+* strain was then transformed with pAT03 to remove the zeocin cassette and make a clean UPAB1 *paaB+* strain. Complemented strains were verified by PCR and sequencing.

### Csu Western blotting and quantification.

Ab17978 overnight cultures were diluted in 10 mL fresh LB to an OD_600_ of 0.05 in 50-mL conical tubes and grown at 37°C with shaking for 4 h until they reached an OD_600_ of 1.0. For growth under TMP/SMX conditions, the concentrations of 3 μg/mL for TMP and 15 μg/mL of SMX were used for Ab17978, and TMP/SMX (1/4 μg/mL) was used for M2. For other antibiotics, bacteria were treated with antibiotic concentrations under which the final culture OD_600_ values were between 65 and 85% of the untreated control. The cells were then pelleted by centrifugation and resuspended in Laemmli buffer to a final OD_600_ of 20. Then, 5 μL of whole-cell samples was loaded onto 15% acrylamide SDS-PAGE gels for separation, transferred to a nitrocellulose membrane, and probed with polyclonal rabbit anti-CsuA/B (1:2,000) (42) and monoclonal mouse anti-RNA polymerase (anti-RNAP) (1:2,600) (BioLegend, San Diego, CA). Western blots were then probed with IRDye-conjugated anti-mouse and anti-rabbit secondary antibodies (both at 1:15,000) (LI-COR Biosciences, Lincoln, NE) and visualized with an Odyssey CLx imaging system (LI-COR Biosciences). Western blot signals were quantified using LI-COR Image Studio software, and the ratio of Csu to RNA polymerase intensity was calculated for at least 4 biological replicates.

### Biofilm assays.

Overnight cultures were normalized to an OD_600_ of 0.05 in LB and inoculated into sterile, round-bottom polystyrene 96-well plates (Corning, Inc.) with a final volume of 200 μL per well. Plates were incubated for 24 h under static conditions at 37°C with humidity to prevent evaporation. After incubation, culture was removed and transferred to another 96-well plate, and OD_600_ readings were taken using a BioTek microplate reader. Wells were washed 5 times with water and stained with 300 μL 0.1% crystal violet solution dissolved in water for 15 min. After staining, wells were washed 5 times with water and left to completely dry overnight. Crystal violet staining was dissolved in 200 μL 30% acetic acid, and absorbance was measured at 550 nm.

### Reverse transcription-quantitative PCR.

Overnight cultures of Ab17978 were inoculated into 50 mL LB or LB plus TMP and SMX (3 μg/mL and 15 μg/mL, respectively) to a final OD_600_ of 0.05 and grown for 2 h at 37°C with shaking. Cells were quickly pelleted and treated with RNAprotect (Qiagen, Inc.), followed by total RNA isolation using the ZR Fungal/Bacterial RNA MiniPrep kit (Zymo Research Corp.). RNA samples were subjected to rigorous DNase treatment using a TURBO DNA-*free* kit to remove contaminating DNA. For reverse transcription (RT)-PCR, cDNA was prepared from 1 μg RNA using a high-capacity RNA-to-cDNA kit (Applied Biosystems), according to the manufacturer’s protocol. The cDNA was diluted 1:10, and 1 μL was used as the template for quantitative PCR (qPCR) using PowerUp SYBR green master mix (Applied Biosystems) on a ViiA7 real-time PCR machine (Applied Biosystems), following the manufacturer’s suggested protocol. The A. baumannii *rpoB* gene was used as a reference gene (41). All gene-specific primers used for qPCR were designed using IDT PrimerQuest and are listed in Table S5. Threshold cycle (*C_T_*) values were normalized to *rpoB*, and fold changes and log_2_(fold changes) were calculated using the ΔΔ*C_T_* method.

### Preparation of whole-cell lysates for comparative proteomics.

Overnight cultures of WT Ab17978 were grown in LB and then subcultured to an OD_600_ of 0.05 in 100 mL of one of the following 4 culture conditions: LB, LB+TMP and SMX (3 μg/mL and 15 μg/mL), LB+PAA (0.5 mM), or LB+PAA+TMP/SMX. Four individual 100-mL culture biological replicates were prepared for each condition. Cultures were grown for 4 h at 37°C with shaking, pelleted, washed 1× with cold phosphate-buffered saline (PBS), and pelleted again. Pellets were flash frozen in liquid nitrogen and stored at −80°C until extraction. For protein extraction, pellets were thawed on ice and resuspended in 25 mL cold PBS plus Pierce protease inhibitor. Cells were lysed with two passages through a cell disruptor at 35,000 lb/in^2^ (Constant Systems, Ltd., Kennesaw, GA). Unbroken cells were pelleted at 8,000 rpm for 10 min. Protein content of lysates was quantified using a DC protein assay kit (Bio-Rad), and 200 μg of protein was acetone precipitated twice. For acetone precipitation, 4 volumes of cold acetone was added to samples, and the samples were incubated overnight at −20°C and centrifuged to discard the supernatant. Protein pellets were resuspended in 200 μL of water, and acetone precipitation was repeated with 4 h of incubation at −20°C. Protein was pelleted at 16,000 × *g* and dried at 70°C for 4 min before being stored at −20°C. No less than 100 μg was used for comparative proteomics.

### Digestion of proteome samples.

Precipitated whole-cell lysates were resuspended in 6 M urea, 2 M thiourea in 40 mM NH_4_HCO_3_ and then reduced for 1 h with 20 mM dithiothreitol (DTT). Reduced samples were then alkylated with 50 mM chloroacetamide for 1 h in the dark. The alkylation reaction was then quenched by the addition of 50 mM DTT for 15 min, and samples were digested with Lys-C (1/200 [wt/wt]) for 3 h at room temperature. Samples were diluted with 100 mM NH_4_HCO_3_ 4-fold to reduce the urea/thiourea concentration below 2 M, then trypsin (1/50 wt/wt) was added, and the mixture was allowed to digest overnight at room temperature. Digested samples were acidified to a final concentration of 0.5% formic acid and desalted with homemade high-capacity StageTips composed of 1 mg Empore C_18_ material (3M) and 5 mg of Oligo R3 reverse-phase resin (Thermo Fisher Scientific) as described previously (54, 55). Columns were wet with buffer B (0.1% formic acid, 80% acetonitrile) and conditioned with buffer A* (0.1% trifluoroacetic acid [TFA], 2% acetonitrile) prior to use. Acidified samples were loaded onto conditioned columns and washed with 10 bed volumes of buffer A*, and bound peptides were eluted with buffer B before being dried then stored at −20°C.

### LC-MS analysis of proteome samples.

Dried proteome digests were resuspended in buffer A* and separated using a two-column chromatography setup composed of a PepMap100 C_18_ 20-mm by 75-μm trap and a PepMap C_18_ 500-mm by 75-μm analytical column (Thermo Fisher Scientific). Samples were concentrated onto the trap column at 5 μL/min for 5 min with buffer A (0.1% formic acid, 2% dimethyl sulfoxide [DMSO]) and then infused into an Orbitrap Fusion Lumos mass spectrometer (Thermo Fisher Scientific) at 300 nL/min via the analytical column using a Dionex Ultimate 3000 UPLC (Thermo Fisher Scientific). One hundred twenty-five-minute analytical runs were undertaken by altering the buffer composition from 2% buffer B (0.1% formic acid, 77.9% acetonitrile, 2% DMSO) to 22% B over 95 min, then from 22% B to 40% B over 10 min, and then from 40% B to 80% B over 5 min. The composition was held at 80% B for 5 min and then dropped to 2% B over 2 min before being held at 2% B for another 8 min. The Orbitrap Fusion Lumos mass spectrometer was operated in a data-dependent mode automatically switching between the acquisition of a single Orbitrap MS scan (300 to 1,600 *m*/*z*, a maximal injection time of 50 ms, an automated gain control [AGC] set to a maximum of 4 × 10^5^ ions, and a resolution of 120,000) and every Orbitrap tandem mass spectrometry (MS/MS) high-energy collisional dissociation (HCD) scan of precursors (normalized collision energy [NCE] of 35%, a maximal injection time of 100 ms, an AGC set to a maximum of 2 × 10^5^ ions, and a resolution of 15,000) every 3 s.

### Proteomic analysis.

Whole-proteome samples were processed using MaxQuant (v1.6.3.4) (56) and searched against the A. baumannii ATCC 17978 proteome (NCBI accession no. CP012004 [3,663 protein sequences]). Searches were undertaken using “trypsin” enzyme specificity with carbamidomethylation of cysteine as a fixed modification. Oxidation of methionine and acetylation of protein N termini were included as variable modifications, and a maximum of 2 missed cleavages were allowed. To enhance the identification of peptides between samples, the “match between runs” option was enabled with a precursor match window set to 2 min and an alignment window of 20 min with the label-free quantitation (LFQ) option enabled (57). The resulting outputs were processed within the Perseus (v1.6.0.7) analysis environment (58) to remove reverse matches and common protein contaminants prior to further analysis. For LFQ comparisons, biological replicates were grouped, and missing values were then imputed based on the observed total peptide intensities with a range of 0.3σ and a downshift of 2.5σ using Perseus. Student *t* tests were undertaken to compare the proteomes between groups, with the resulting data exported and visualized using ggplot2 (59) within R. The resulting MS data and search results have been deposited into the PRIDE ProteomeXchange Consortium repository (60, 61).

### Preparation of samples for RNA sequencing.

WT Ab17978 and Ab17978 Δ*paaB* were grown overnight in LB before being diluted into 10 mL of LB or LB+TMP/SMX (3 μg/mL and 15 μg/mL, respectively) and grown for 2 h at 37°C with shaking. Four individual overnight and 10-mL culture biological replicates were prepared for each condition. Cultures were normalized, and the equivalent of 1 mL of an OD_600_ of 1.0 was pelleted quickly at 8,000 rpm and treated with RNAprotect for 5 min at room temperature. Pellets were flash frozen and stored at −80°C until extraction. For RNA extractions, pellets were thawed on ice, resuspended in 600 μL TRIzol (Invitrogen) with 4 μL of glycogen at 5 mg/mL, and lysed via bead beating. Samples were pelleted, and supernatants were treated with chloroform. RNA was extracted from the aqueous phase using the RNeasy minikit (Qiagen, Inc.), and RNA quality was checked by agarose gel electrophoresis and *A*_260_/*A*_280_ measurements. RNA was stored at −80°C with SUPERase-IN RNase inhibitor (Life Technologies) until library preparation.

### RNA sequencing and analysis.

RNA sequencing (RNA-Seq) was performed as done previously (62). Briefly, 400 ng of total RNA from each sample was used for generation of cDNA libraries following the RNAtag-Seq protocol. PCR-amplified cDNA libraries were sequenced on an Illumina NextSeq500, generating a high sequencing depth of ~7.5 million reads per sample. Raw reads were demultiplexed by 5′ and 3′ indices, trimmed to 59 bp, and quality filtered (96% sequence quality > *Q*14). Filtered reads were mapped to the corresponding reference genome using bowtie2 with the “very-sensitive” option (-D 20 –R 3 –N 0 –L 20 –i S, 1, 0.50). Mapped reads were aggregated by feature Count, and differential expression was calculated with DESeq2 (63, 64). In each pairwise differential expression comparison, significant differential expression was filtered based on two criteria: |log_2_ fold change| of >1 and adjusted *P* value (padj) of <0.05. All differential expression comparisons were made between the WT and *paaB* mutant either in LB or under TMP/SMX treatment. The reproducibility of the transcriptomic data was confirmed by an overall high Spearman correlation across biological replicates (*R* > 0.95). Raw RNA-Seq data sets are available at the Sequence Read Archive (BioProject accession no. PRJNA726989). Differential expression data can be found in Tables S2 to S3 and at https://www.ncbi.nlm.nih.gov/Traces/study/?acc=PRJNA726989&o=acc_s%3Aa.

### Growth assays.

Growth curves were performed in sterile, round-bottom, polystyrene, 96-well plates. Bacterial strains were grown overnight in LB, washed in PBS, and diluted in fresh medium (LB or M9 minimal medium supplemented with PAA derivatives) to an OD_600_ of 0.01. Bacterial suspensions were then inoculated into 96-well plates at a 150-μL final volume before being grown at 37°C under shaking conditions. OD_600_ values were measured at 30-min intervals for 16 h with a BioTek microplate reader. For antibiotic growth curves, the same protocol was used, and bacteria were diluted in LB with the appropriate antibiotic concentration. All experiments were performed on 3 independent days with at least 3 wells per strain per condition.

### Drug susceptibility assays (MIC determination).

Antimicrobial susceptibility was determined using the 2-fold broth dilution microtiter assay as previously described (32, 65). Briefly, cells were inoculated at a density of 10^5^ cells/mL in a 96-well microtiter plate containing 2-fold dilutions of the appropriate antibiotics. MICs were visually determined after plates were incubated at 37°C under shaking conditions for 16 h.

### H_2_O_2_ killing assays.

H_2_O_2_ killing assays were performed as done previously (38). Briefly, overnight cultures of the WT, Δ*paaB*, and *paaB+* UPAB1 strains were diluted 1:1,000 into 10 mL LB in 50-mL conical tubes and grown to mid-exponential phase (OD_600_ of 0.4 to 0.6). Cultures were then treated with 0 mM or 175 mM H_2_O_2_ for 30 min before being serially diluted and spot plated onto LB agar to enumerate CFU. Experiments were performed at least 3 independent times and were plated in duplicate.

### Murine CAUTI model.

CAUTI infections were performed as previously described for A. baumannii (37). Bacterial strains were prepared for inoculation after static growth twice at 37°C by centrifugation at 6,500 rpm for 5 min. Cells were washed twice in 1× PBS and resuspended in PBS to the final inoculum. Briefly, 6- to 8-week-old female C57BL/6 mice (Charles River Laboratories) were anesthetized by inhalation of 4% isoflurane, and a 4- to 5-mm piece of silicone tubing (catheter) was placed in the bladder via transurethral insertion. Mice were infected immediately following implant placement with ~2 × 10^8^ CFU bacteria in 50 μL via transurethral inoculation. At 24 h postinfection (p.i.), mice were euthanized, and bladders and catheters were aseptically removed. The bacterial load present in each tissue was determined by homogenizing each organ in PBS and plating serial dilutions on LB agar supplemented with antibiotics when appropriate. All CAUTI studies were performed in accordance with the guidelines of the Committee for Animal Studies at Washington University School of Medicine, and we have complied with all relevant ethical regulations. Mice were housed with a cycle consisting of 12 h each of light and dark with access to standard food and water *ad libitum*.

### Statistical analyses.

Statistical analyses were performed using GraphPad Prism 9 software (GraphPad Software, Inc., La Jolla, CA).

### Data availability.

The mass spectrometry proteomics data have been deposited into the PRIDE ProteomeXchange Consortium repository and can be accessed with the identifier PXD025651. Raw RNA-Seq data sets are available at the Sequence Read Archive (BioProject accession no. PRJNA726989).
