# Supplementary figures and images for: The Phenylacetic Acid Catabolic Pathway Regulates Antibiotic and Oxidative Stress Responses in Acinetobacter
Source: mBio. 2022 Apr 25;13(3):e01863-21. doi: 10.1128/mbio.01863-21 (PMC9239106; doi:10.1128/mbio.01863-21)

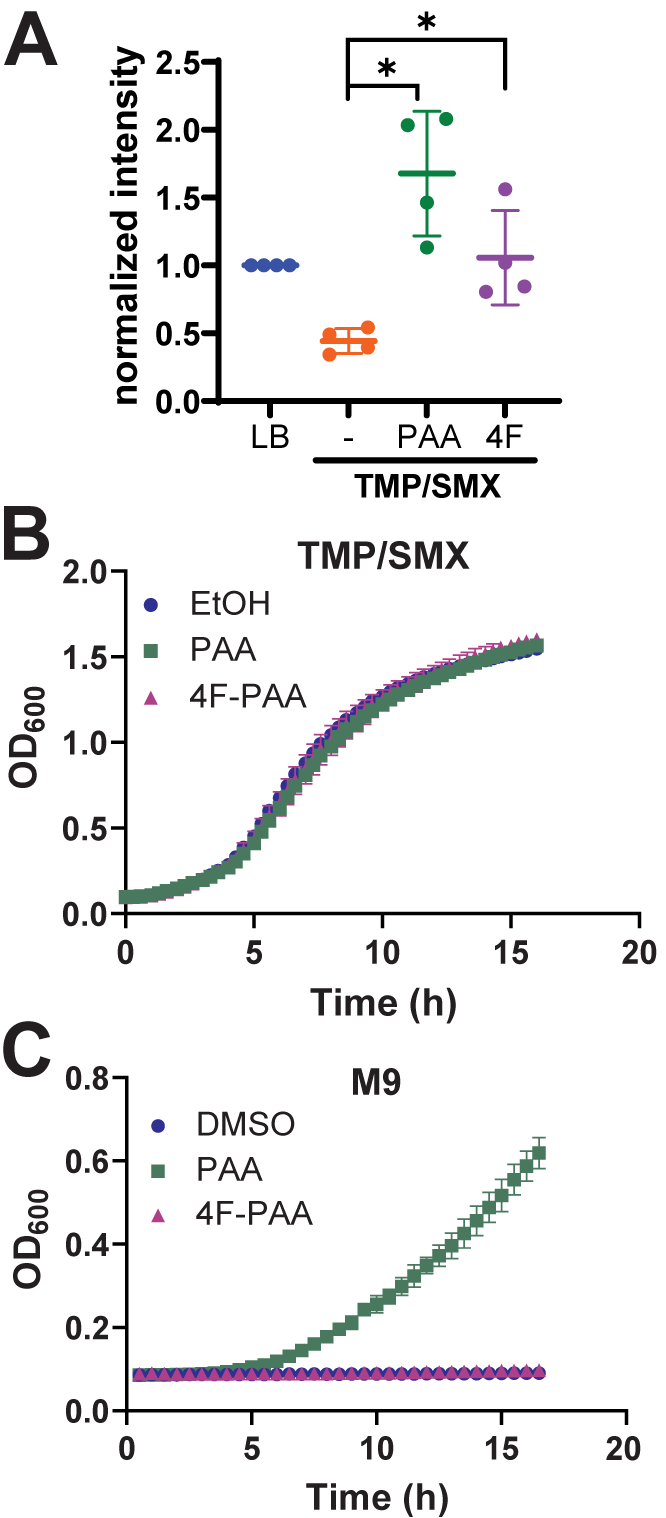

Supplement: FIG S1 [file mbio.01863-21-sf001.tif]

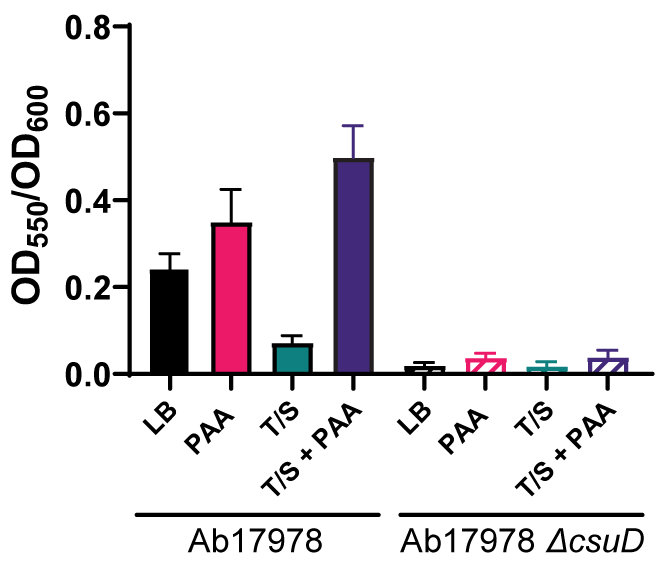

Supplement: FIG S2 [file mbio.01863-21-sf002.tif]

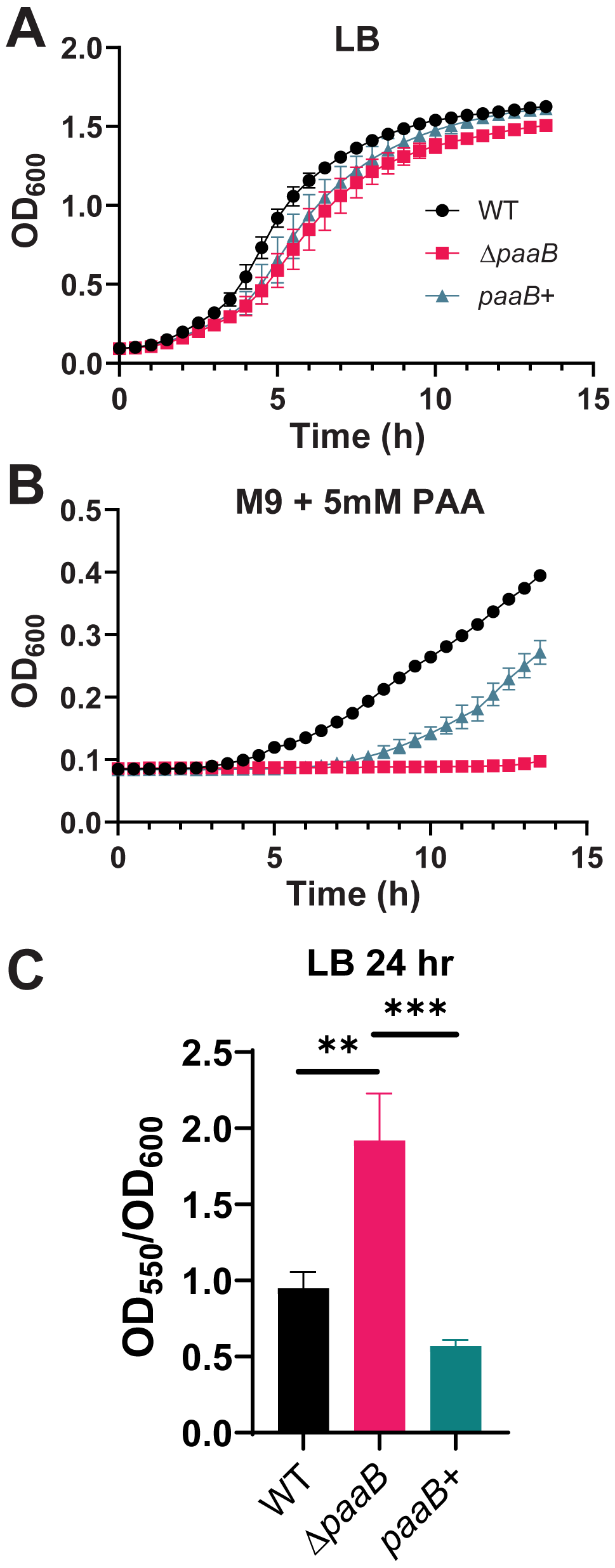

Supplement: FIG S3 [file mbio.01863-21-sf003.tif]

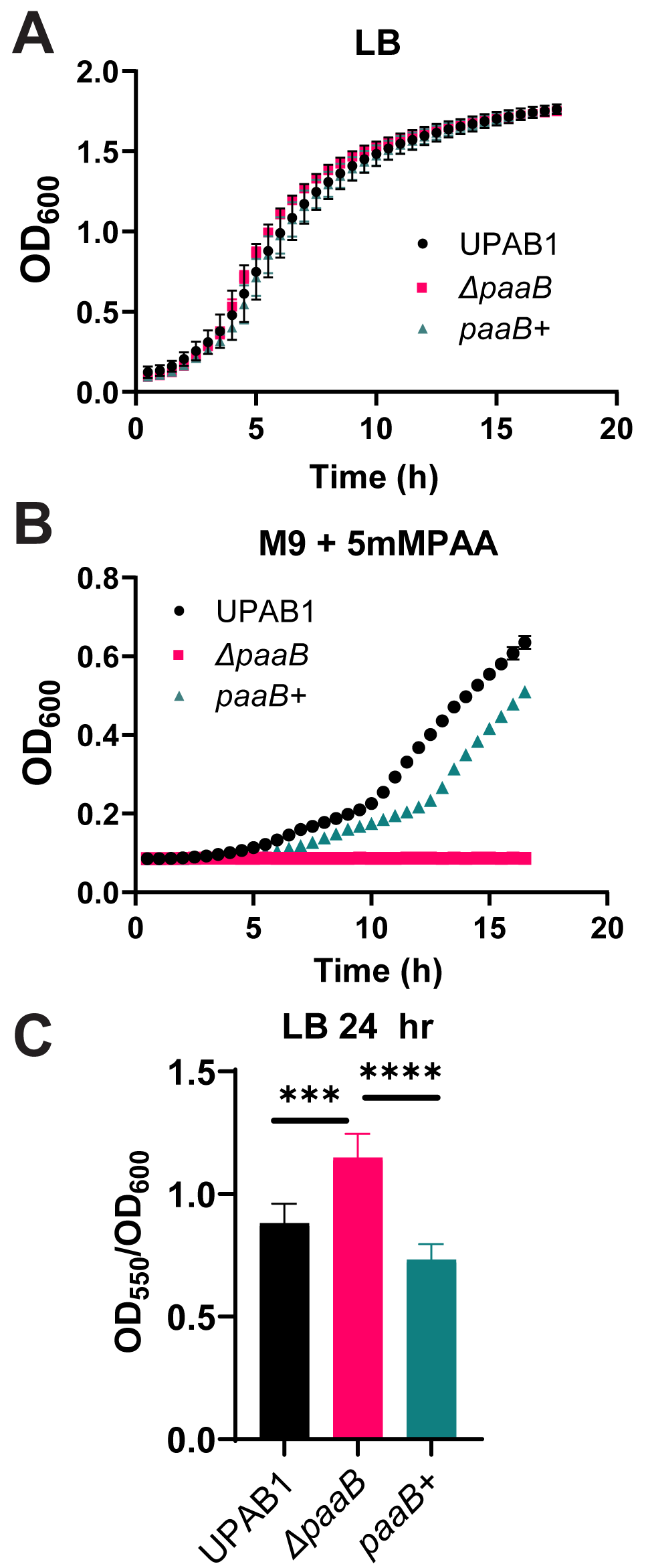

Supplement: FIG S4 [file mbio.01863-21-sf004.tif]
