# Supplementary material for: The Phenylacetic Acid Catabolic Pathway Regulates Antibiotic and Oxidative Stress Responses in Acinetobacter
Source: mBio. 2022 Apr 25;13(3):e01863-21. doi: 10.1128/mbio.01863-21 (PMC9239106; doi:10.1128/mbio.01863-21)
Supplement: TABLE S2 [file mbio.01863-21-st002.pdf]

Supplementary Table 2. Differentially regulated chromosomal genes in *ΔpaaB* v. WT in LB

Gene	Annotated function of encoded protein	Fold Change
ACX60_RS03460	entericidin A/B family lipoprotein	-2.03
ACX60_RS05035	hypothetical protein	-2.06
ACX60_RS06110	HemP - hemin uptake protein	-2.53
ACX60_RS06330	30S ribosomal protein S21	-2.48
ACX60_RS06605	hypothetical protein	-2.97
ACX60_RS06820	3-hydroxyacyl-CoA dehydrogenase	-2.12
ACX60_RS09200	GntP - family permease	-2.44
ACX60_RS09235	CoA transferase subunit A	-2.81
ACX60_RS09240	CoA transferase subunit B	-3.01
ACX60_RS09245	short chain fatty acid transporter	-2.93
ACX60_RS10515	hypothetical protein	-2.72
ACX60_RS10740	hypothetical protein	-2.41
ACX60_RS11185	stress-induced protein	-2.15
ACX60_RS11500	AbaF - fosfomycin efflux transporter	-3.23
ACX60_RS11805	YcsF-family protein	-2.19
ACX60_RS12200	KTSC domain-containing protein	-2.07
ACX60_RS12960	hypothetical protein	-2.06
ACX60_RS16165	CrcB- fluoride efflux transporter	-2.59
ACX60_RS17775	EpsG family protein	-2.08
ACX60_RS17880	ferredoxin reductase	-2.13
ACX60_RS18150	GlsB/YeaQ/YmgE family stress response protein	-2.14
ACX60_RS19405	hypothetical protein	-2.04
ACX60_RS02105	RplW - 50S ribosomal protein	2.30
ACX60_RS03825	AdeK	2.84
ACX60_RS03830	AdeJ	3.59
ACX60_RS03835	AdeI	2.95
ACX60_RS03840	phosphatase PAP2 family protein	2.88
ACX60_RS11415	PaaI	2.43
ACX60_RS11420	PaaY	2.15
ACX60_RS11425	PaaX	3.05
ACX60_RS11430	PaaK	2.57
ACX60_RS11435	PaaJ	3.05
ACX60_RS11440	PaaH	3.67
ACX60_RS11445	PaaG	3.28
ACX60_RS11450	PaaF	3.28
ACX60_RS11455	PaaE	2.41
ACX60_RS11460	PaaF	3.29
ACX60_RS11465	PaaC	3.12
ACX60_RS11475	PaaA	6.13
ACX60_RS11480	PaaZ	5.14
ACX60_RS14645	hypothetical protein	2.20
