# Supplementary material for: The Phenylacetic Acid Catabolic Pathway Regulates Antibiotic and Oxidative Stress Responses in Acinetobacter
Source: mBio. 2022 Apr 25;13(3):e01863-21. doi: 10.1128/mbio.01863-21 (PMC9239106; doi:10.1128/mbio.01863-21)
Supplement: TABLE S3 [file mbio.01863-21-st003.pdf]

Supplementary Table 3. Differentially regulated chromosomal genes in *ΔpaaB* v. WT in TMP/SMX

Gene	Annotated function of encoded protein	Fold Change
ACX60_RS00260	DUF1328 domain-containing protein	-2.21
ACX60_RS00325	HppD - 4-hydroxyphenylpyruvate dioxygenase	-3.13
ACX60_RS00335	homogentisate 1,2 dioxygenase	-5.69
ACX60_RS00340	MaiA - maleylacetoacetate isomerase	-4.52
ACX60_RS00345	FahA - fumarylacetoacetase	-6.79
ACX60_RS00350	amino acid permease	-3.05
ACX60_RS00430	hypothetical protein	-2.19
ACX60_RS00695	ribosome-associated protein	-2.08
ACX60_RS01850	hypothetical protein	-2.48
ACX60_RS05490	hypothetical protein	-2.06
ACX60_RS06110	HemP - hemin uptake protein	-2.07
ACX60_RS06425	YdeI family stress tolerance protein	-2.01
ACX60_RS06605	hypothetical protein	-2.72
ACX60_RS07415	hypothetical protein	-2.03
ACX60_RS07420	hypothetical protein	-2.12
ACX60_RS09235	CoA transferase subunit A	-2.14
ACX60_RS10070	HNH endonuclease	-2.01
ACX60_RS10740	hypothetical protein	-2.01
ACX60_RS11185	stress-induced protein	-2.20
ACX60_RS11500	AbaF - fosfomycin efflux transporter	-3.17
ACX60_RS12065	hypothetical protein	-2.76
ACX60_RS12910	gamma-aminobutyraldehyde dehydrogenase	-2.19
ACX60_RS14780	acyl-CoA dehydrogenase	-2.48
ACX60_RS15630	glycosyltransferase family 1 protein	-2.63
ACX60_RS15900	HdeD family acid-resistance protein	-2.12
ACX60_RS16165	CrcB - fluoride efflux transporter	-2.21
ACX60_RS16640	hypothetical protein	-2.35
ACX60_RS16810	EamA family transporter	-2.31
ACX60_RS17680	DUF4126 domain-containing protein	-2.11
ACX60_RS17775	EpsG family protein	-2.46
ACX60_RS17930	sulfonate ABC transporter substrate-binding protein	-2.27
ACX60_RS03825	AdeK	2.96
ACX60_RS03830	AdeJ	3.51
ACX60_RS03835	AdeI	3.11
ACX60_RS03840	phosphatase PAP2 family protein	2.85
ACX60_RS05510	metal dependent hydrolase	2.04
ACX60_RS05950	septal ring lytic transglycosylase	2.19
ACX60_RS06470	hypothetical protein	2.04
ACX60_RS06475	TetR family transcriptional regulator	3.52
ACX60_RS06480	CsuA/B	4.11
ACX60_RS06485	CsuA	4.83
ACX60_RS06490	CsuB	6.45
ACX60_RS06495	CsuC	4.21

ACX60_RS06500	CsuD	3.90
ACX60_RS06505	CsuE	2.74
ACX60_RS06640	Fis - DNA binding transcriptional regulator	2.03
ACX60_RS07860	hypothetical protein	2.09
ACX60_RS08350	phosphopyruvate hydratase	2.00
ACX60_RS09275	AspA - aspartate ammonina lyase	2.28
ACX60_RS10395	cupin domain-containing protein	2.06
ACX60_RS11425	PaaX	2.22
ACX60_RS11430	PaaK	2.08
ACX60_RS11435	PaaJ	2.05
ACX60_RS11440	PaaH	2.43
ACX60_RS11445	PaaG	2.38
ACX60_RS11450	PaaF	2.73
ACX60_RS11455	PaaE	2.94
ACX60_RS11460	PaaD	3.26
ACX60_RS11465	PaaC	3.06
ACX60_RS11475	PaaA	3.10
ACX60_RS11480	PaaZ	3.55
ACX60_RS12905	AsnC family transcriptional regulator	2.26
