# Supplementary material for: The Phenylacetic Acid Catabolic Pathway Regulates Antibiotic and Oxidative Stress Responses in Acinetobacter
Source: mBio. 2022 Apr 25;13(3):e01863-21. doi: 10.1128/mbio.01863-21 (PMC9239106; doi:10.1128/mbio.01863-21)
Supplement: TABLE S4 [file mbio.01863-21-st004.pdf]

Supplementary Table 4. Bacterial strains and plasmids used in this study.

Strain	Description/plasmids
*Ab*17978	*A. baumannii* ATCC 17978 with pAB3 plasmid from (1)
M2	*A. nosocomialis* strain (2)
UPAB1	Uropathogenic *A. baumannii* clinical isolate from (3)
*Ab*17978 *ΔpaaB*	Ab17978 with clean deletion of *paaB*
*Ab*17978 *paaB*+	*Ab*17978 *ΔpaaB* with chromosomal mTn7 insertion of *paaABC* with its 300bp upstream region
*Ab*17978 *ΔcsuD*	Ab17978 Csu mutant from (4)
UPAB1 *ΔpaaB*	UPAB1 with clean deletion of *paaB*
UPAB1 *paaB*+	UPAB1 *ΔpaaB* with chromosomal mTn7 insertion of *paaAB* with its 300bp upstream region
E. coli TOP10	*E. coli* strain containing pUCT18-miniTn7-Gm-*paaABC*
E. coli TOP10	*E. coli* strain containing pUCT18-miniTn7-Zeo-*paaAB*
**Plasmid**	**Description**
pUCT18-miniTn7-Gm-*paaABC*	*paaABC* with 300bp upstream cloned from Ab17978 into pUCT18-miniTn7- Gm from from (5)
pUCT18-miniTn7-Zeo-*paaAB*	*paaAB* with 300bp upstream cloned from UPAB1 into pUCT18-miniTn7-Zeo from (6)

References

1. Weber BS, Ly PM, Irwin JN, Pukatzki S, Feldman MF. 2015. A multidrug resistance plasmid contains the molecular switch for type VI secretion in *Acinetobacter baumannii*. Proc Natl Acad Sci 112:9442–9447.
2. Carruthers MD, Nicholson PA, Tracy EN, Munson RS. 2013. Acinetobacter baumannii Utilizes a Type VI Secretion System for Bacterial Competition. PLoS One 8.
3. Di Venanzio G, Flores-mireles AL, Calix JJ, Haurat MF, Scott NE, Palmer LD, Potter RF, Hibbing ME, Friedman L, Wang B, Dantas G, Skaar EP, Hultgren SJ, Feldman MF. 2019. Urinary tract colonization is enhanced by a plasmid that regulates uropathogenic Acinetobacter baumannii chromosomal genes. Nat Commun 10:1–13.
4. Moon KH, Weber BS, Feldman F. 2017. Subinhibitory Concentrations of Trimethoprim and Sulfamethoxazole Acinetobacter baumannii through Inhibition of Csu Pilus Expression. Antimicrob Agents Chemother 61:1–18.
5. Kumar A, Dalton C, Cortez-Cordova J, Schweizer HP. 2010. Mini-Tn7 vectors as genetic tools for single copy gene cloning in Acinetobacter baumannii. J Microbiol Methods 82:296–300.
6. Ducas-Mowchun K, De Silva PM, Crisostomo L, Fernando DM, Chao TC, Pelka P, Schweizer HP, Kumar A. 2019. Next generation of Tn7-based single-copy insertion elements for use in multi- and pan-drug-resistant strains of Acinetobacter baumannii. Appl Environ Microbiol 85:1–10.
