# Supplementary material for: The Phenylacetic Acid Catabolic Pathway Regulates Antibiotic and Oxidative Stress Responses in Acinetobacter
Source: mBio. 2022 Apr 25;13(3):e01863-21. doi: 10.1128/mbio.01863-21 (PMC9239106; doi:10.1128/mbio.01863-21)
Supplement: TABLE S5 [file mbio.01863-21-st005.pdf]

Supplementary Table 5. Oligos used in this study.

Name	Sequence
P1	AGCGATTGTGTAGGCTGGAGCTG
P2	CATATGAATATCCTCCTTAGTTCCTATTCCG
UP paaB 5' Fwd	GTCGATAACACTGTACCGCAAG
UP paaB-zeo 5' Rev	GCAGCTCCAGCCTACACAATCGCTCCATAAATCTTGTTAAGCCAC
UP zeo-paaB 3' Fwd	GAACTAAGGAGGATATTCATATGGGGAATGGCAAATGAATCATTC
UP paaB 3' Rev	CAACCACTGGCGTACAATCG
UPAB1 paaB flank F	GAAGCCATGATGGCTTTGGC
UPAB1 paaB flank R	GATTTCACAGCAAGCGCG
17 paaBKO Fwd	AATGGGTTCGTGATGCAGCAGTGGTCTACGCGAAAAAACAGCAGCTTGAAGCG AACAAAGTGGCTTAACAAGATTTATGGAAAAATTTGAGGAAATCAAAATGGAACA TATGAATATCCTCCTTAGTTCCTATTCCG
17 paaBKO Rev	ATGCCCGCACCATTCAGCCAAACGCTGAGATAAAACCAGTTGGCTGTCGCCAAT ATGTAATAAGAATTTAGATAAAACTGAATGATTCATTTGCCATTCCCCTTACATAG CGATTGTGTAGGCTGGAGCTGCTTCG
17 paaB flank F	ATCGTTTGTGCAAAGTCACC
17 paaB flank R	AACTTGGCTTGGAAGTACCG
17paaX 5’ Fwd	GCTCCATATATTCTTGCGTGCC
17paaX-frt 5’ Rev	CGAAGCAGCTCCAGCCTACACAATCGCTCATGCCTTGTTATAGTATTGA CGG
17paaX-frt 3’ Fwd	CGGAATAGGAACTAAGGAGGATATTCATATGACTCATGTCTTGGCGTTT AGTTTCAGG
17paaX 3’ Rev	GAAGGCTTACCGATTATTCGTTACCG
17 paaX flank F	CTCTTGCATTGAATTTAAGCAGCGC
17 paaX flank R	CAGGTGAAGTCTGTAAAGATGGCG
17 pUCT-paaABC Fwd	CATGCATGAGCTCACTAGTGGATCCCTCACCTCCTTGTCTTACCAGG
17 pUCT-paaABC Rev	GCGAGGTACCGGGCCCAAGCTTGTAATTCTTCGTCAGACGAATGCC
UP pUCT-paa operon Fwd	CATGAGCTCACTAGTGGATCCCTGTGTCAGTTGAGGTTCCTTCTAGG
UP pUCT-paa operon Rev	GCGAGGTACCGGGCCCAAGCTTTTAGCGCCCTTTAAAATTTGGTTCACG
UPAB1 paaB Rev	GCCATTCCCCTTACATGTGC
rpoB FWD set 5	ACGGTACTGAGCGTGTAATC
rpoB REV set 5	TTACCACTTGAGTGGGTCTTAC
csuA/B FWD set 1	GTTGCTGGTTATGCGGTAAATA
csuA/B REV set 1	TCAGTTTGACTACCACCTACA
paaA FWD Set 6	TCTCGCCAAACTCATAGTGG
paaA REV Set 6	GTCGATAACACCGTACCTCAA
paaB FWD set 2	AGCTTAAGAGCACCTGATGAT
paaB REV set 2	CTGAACGAACCACCCAAATG
mao FWD Set 6	ACTTATTCCAGGTGGCTCTTC
mao REV Set 6	CAGCGGGCATACGAATAGA
feaB FWD Set 7	CAAACCATGCAACCCTCTATTC
feaB REV Set 7	ATTCCAAGGCACAATCCCT
